# Boronize Coatings Studied with a New Mass Transfer Model

**DOI:** 10.3390/ma17215309

**Published:** 2024-10-31

**Authors:** Ángel Jesús Morales-Robles, Martín Ortiz-Domínguez, Oscar Armando Gómez-Vargas, María de la Luz Moreno-González

**Affiliations:** 1 Academic Area of Earth Sciences and Materials, Institute of Basic Sciences and Engineering, Autonomous University of Hidalgo State, Carretera Pachuca-Tulancingo Km. 4.5 s/n, Colonia Carboneras, Mineral de la Reforma 42184, Hidalgo, Mexico; mo298963@uaeh.edu.mx; 2 Mechanical Engineering, Escuela Superior de Ciudad Sahagún, Autonomous University of Hidalgo State, Carretera Ciudad Sahagún-Otumba s/n, Zona Industrial, Ciudad Sahagún 43990, Hidalgo, Mexico; 3 Division of Graduate Studies and Research, Tlalnepantla Institute of Technology, National Technological Institute of Mexico (ITTLA/TecNM), Avenida Instituto Tecnológico, s/n, Colonia La Comunidad, Tlalnepantla de Baz 54070, Estado de México, Mexico; oscar.gv@tlalnepantla.tecnm.mx; 4 Industrial Engineering Department, Tlalnepantla Institute of Technology, National Technological Institute of Mexico (ITTLA/TecNM), Avenida Instituto Tecnológico, s/n, Colonia La Comunidad, Tlalnepantla de Baz 54070, Estado de México, Mexico; maria.mg@tlalnepantla.tecnm.mx

**Keywords:** thermochemical surface technique, hardening process, hard coating, mass transfer model, potential barrier, wear, ANOVA analysis

## Abstract

This study examined the development of Fe_2_B (diiron boronize) coatings on the surface of 35NiCrMo4 steel through the thermochemical surface hardening process called boronizing. The morphology and thickness of the boronize coatings were assessed using Scanning Electron Microscopy (SEM) and optical microscopy (OM). A novel mathematical mass transfer model was developed to estimate the diffusion coefficients of boron in hard coating. The presence of uniformly distributed boronize coatings with a typical sawtooth pattern on the surface of the substrate was confirmed. The boronize coating’s chemical composition and phase constituents were analyzed utilizing X-ray energy dispersive spectroscopy (EDS) and X-ray diffraction analysis (XRD). The study confirmed the presence of a single-phase boronize coating (Fe_2_B). Furthermore, microhardness tests indicated that the boronized specimen’s surface demonstrated an average hardness of approximately 1953 HV. The wear study were conducted using the pin-on-disk method under dry debonding conditions at room temperature to estimate the coefficient of friction (COF) of the boronized (average ≈ 0.35) and untreated (0.725) specimens. The results revealed approximately 200% improvement in wear resistance due to the boronized coating. The empirical validation of the mathematical model was carried out for two additional boronizing conditions at 1223 K for 3 h and 1273 K for 1.5 h, resulting in an estimated percentage error of around 2.5% for both conditions. Additionally, an ANOVA analysis was performed, taking into account the temperature and time factors. The findings indicate that both factors exert a substantial influence on the dependent variable (*u*), with temperature (*T*) contributing 64.68%, time (*t*) contributing 27.37%, and the interaction of both factors (*T* × *t*) contributing 5.13%.

## 1. Introduction

The durability of various engineering components is often contingent upon the surface properties of the materials. The predominant sought-after characteristics in industrial applications include wear, corrosion, and oxidation resistance [1]. A wide array of coatings is employed to enhance the performance of materials and safeguard metal surfaces from environmental exposure. Surface modification through the formation of compounds resulting from the diffusion of chemical elements that react with the substrate is a widely employed practice in various industrial applications. This method involves the generation of surface coatings via the diffusion of carbon, nitrogen, boron, and sulfur atoms, individually or in combination with other metallic elements. These processes are facilitated through the thermal activation of the metal, resulting in the desired surface modifications. As a result, the material’s surface forms new phases with distinct mechanical and chemical properties. Surface modification techniques such as carburizing, nitriding, and boronizing are notable [1]. Boronizing is a metallurgical surface-hardening process applicable to a wide range of metals, encompassing nearly all metallic elements in the periodic table, with the exception of aluminum, copper, and magnesium [2].

Boronizing is a surface treatment process that yields markedly higher hardness values in steels than carburizing and nitriding. This is attributed to the creation of high-hardness iron boronizes on the surface. These boronizes form a continuous and compact phase that is chemically more stable than the steel substrate. They exhibit a low friction coefficient, effectively reducing wear [3]. In high-stakes industrial applications, such as those involving oil well casing, the significant abrasive wear and consequential high replacement costs have prompted a growing interest in high-performance surface hardening processes. For instance, the boronizing process has been effectively employed to enhance the lifetime of these pipes. Similarly, critical components like bearings in wind power generators have shown potential for replacing the carburizing process with the boronizing process for improved performance [4,5]. Boronizing represents a relatively underexplored thermochemical treatment compared to other solid-state diffusion hardening processes such as carburizing and nitriding. This can be attributed, in part, to the requirement for relatively high temperatures (1123–1273 K) over extended periods of 2, 4, 6, and 8 h. These conditions lead to notable alterations in the microstructure of steels, including grain growth and a subsequent reduction in mechanical properties.

Obtaining boronize coatings with a thickness adequate to the service demand at temperatures that do not cause grain growth depends on the efficiency of boron diffusion, which is why the process continues to be investigated. To this end, several approaches published by different authors were cited to estimate the growth diffusion of hard coatings formed on ferrous alloys with or without a consideration of iron boronize incubation times. In their work, Elías-Espinosa et al. [6] developed a diffusion model to analyze the growth kinetics of AISI O1 steel through the solid boronizing treatment. The study focused on applying the balance principle or conservation of mass at the growth interface (diiron boronize coating/Fe) to address the diffusion problem, considering the characteristic incubation time for iron boronize. The researchers determined the potential barrier (or activation energy) for the diffusion of boron atoms in AISI O1 steel to be 197.2 kJ mol^−1^. Kouba et al. [7] introduced an alternative method for analyzing the growth diffusion of boronized ARMCO^®^ pure iron. Their model utilized the surface boron flux as a fitting parameter to replicate the observed values of boronize incubation times during the forming of Fe_2_B coatings. Similarly, Hu et al. [3] examined the growth kinetics of a high-entropy CoCrFe-NiMn alloy, employing a straightforward model that assumed the parabolic growth law for iron boronizes. Using the parabolic growth law, Ulloa et al. [8] investigated boron diffusion in an 80/20 nickel–chromium alloy.

This study aimed to conduct boronizing on 35NiCrMo4 steel at various temperatures and treatment durations under controlled atmospheric conditions using a commercial boron mixture comprising 35 wt.% boron carbide, 5.4 wt.% potassium tetrafluoroborate, and 61.1 wt.% silicon carbide. A new diffusion model was employed to determine the boron diffusivity in diiron boronize coatings on the substrate. Furthermore, the activation energy of boron was calculated using this model. A comparative analysis was performed with data from previous studies, followed by an assessment of the resultant changes in the surface properties of the substrate post-boronizing. In this study, microstructural characterization employed OM and SEM methods, while chemical characterization of the boronize coatings resulting from the boronizing process utilized XRD and EDS methods. The microindentation hardness testing of the hard coatings was conducted by applying the Vickers hardness method. The study examined the boronizing process’s impact on the base material’s tribological characteristics. Wear tests were conducted using the pin-on- disk method during dry sliding conditions at the average temperature of an environment to assess this impact. Additionally, Rockwell-C indentation adhesion tests were employed. Subsequently, analysis of variance (ANOVA) was used to ascertain the impact of temperature and time. In addition, a simple regression approach was carried out to derive 35NiCrMo4 steel as a function of the independent variables. Digital methods were used to analyze the SEM micrographs from the growth fronts of the diiron boronize coatings on the surface of 35NiCrMo4 steel. The analysis aimed to determine the total area of the Fe_2_B phase and the accumulated area relative to the penetration depth (µm).

## 2. Materials and Methods

### Materials and Thermochemical Treatment

**Figure 1:**
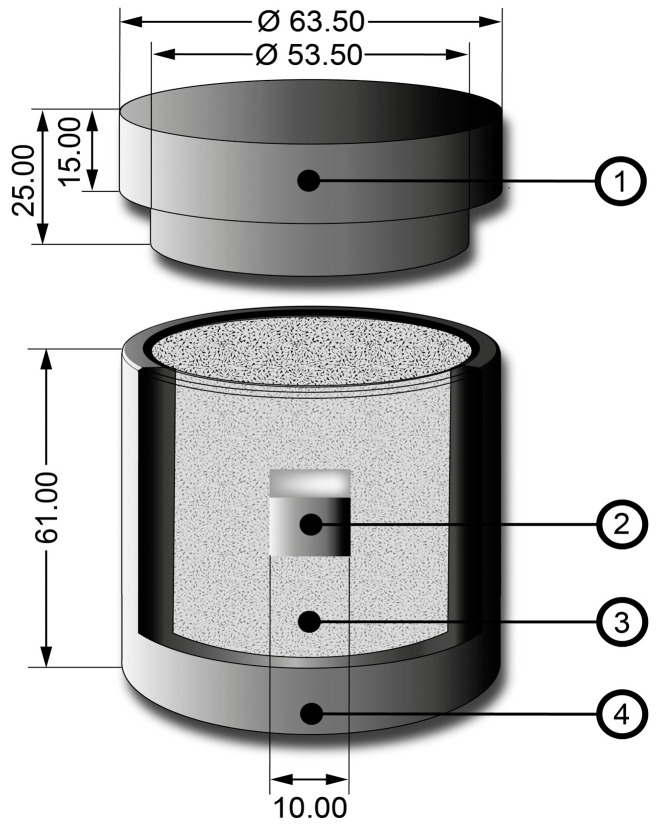
A cylindrical AISI 316L stainless steel container used for the thermochemical surface hardening process is presented in a schematic illustration and cross-sectional view. The container comprises four key components: ① a lid featuring a gas release orifice to manage the byproduct gases from reaction of the components that make up the boron-rich mixture; ② the substrate; ③ Boron carbide, Potassium tetrafluoroborate, and Silicon carbide; and ④ the cylindrical container itself. The container’s dimensions are provided in millimeters.

35NiCrMo4 steel is a machinery grade or low-alloy steel with the following composition: 0.38–0.43% carbon, 0.70–0.90% manganese, 0.85–1.15% nickel, 0.20–0.30% molybdenum, 0.15–0.35% silicon, 0.70–0.90% chromium, 0.10–0.30% vanadium, 0.040% phosphorus, and 0.040% sulfur. This type of steel possesses improved hardenability and can be hardened by quenching and tempering to achieve hardness values of around 540 HV when in an annealed condition. It is commonly utilized in the production of mechanical parts for machinery, including fastening and motion transmission elements such as shafts, low-speed gears, worm gears, screws, high-strength bolts, machine bodies, and torsion bars. The specimens to be processed were cubic, with dimensions of 10 mm on each side. They were obtained by sectioning a square bar using a Buehler-IsoMetTM 1000 (Lake Bluff, IL, USA) precision gravity bench. Before the thermochemical treatment, the steel specimens underwent surface roughening using silicon carbide (SiC) sandpaper with particle sizes ranging from 80 to 2500, applied using an EcoMetTM 30 (Lake Bluff, IL, USA) single-hand grinding machine. Subsequently, the specimens were subjected to a 20-min treatment in a SONOREX SUPER RK 52 (Heinrichstraße, Berlín, Germany) high-performance ultrasonic bath using a binary liquid solution comprising n-heptane and ethanol. After washing, the specimens were confined in a cylindrical AISI 316L stainless steel recipient and immersed in a mixture of boron-rich powder, as illustrated in Figure 1.

The powder mixture utilized in the thermochemical treatment comprised 33.5% B_4_C, 61.1% SiC, and 5.4% KBF_4_. Subsequent to its melting point, KBF_4_ is likely to decompose into KF and BF_3_ [9].

(1) $$KBF_{4}\to BF_{3}+KF.$$

Volatile boron halides (BF_2_, BF) transport B to the substrate surface. The BF_2_ gas dissociates into B and [BF_3_], and the chemical reaction proceeds with the diffusion of B^+^ ions on the steel surface. The chemical reactions that form diiron boronize coatings are described below:

(2) $$2BF_{3}+B_{4}C\to 3BF_{2}+3\left[B\right]+C,$$

(3) $$3BF_{2}\to \left[B\right]+2BF_{3},$$

(4) $${\left[B\right]}^{+}+2Fe\to Fe_{2}B.$$

**Figure 2:**
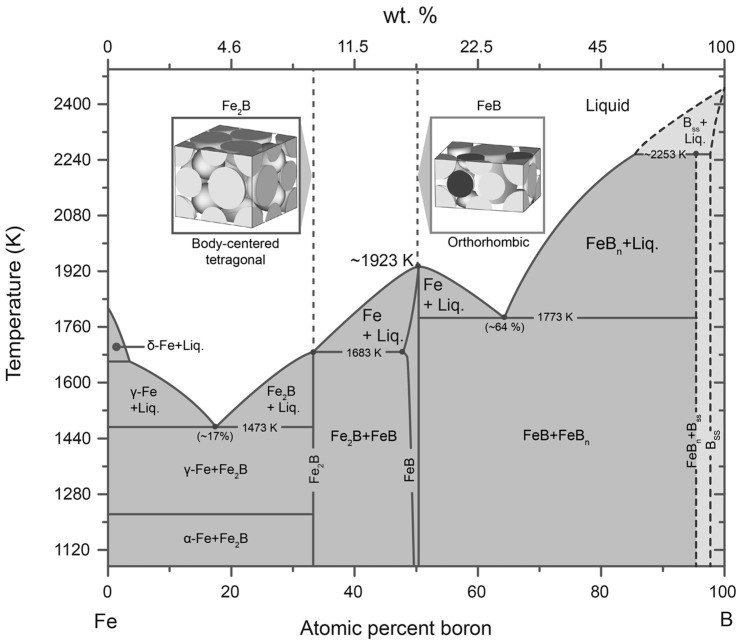
Illustration of the Fe-B phase diagram, with the crystal structures of the boron phases respectively.

The process was conducted using a TEFIC 1473 K furnace (Distrito de Weiyang, Xi’an, China), with varying treatment times and temperatures under the protection of argon gas to prevent atmospheric contamination by oxygen (O) and hydrogen (H). Argon (Ar) gas, being chemically inert, tasteless, and odorless, was the ideal choice for this purpose. The AISI 316L steel vessel was heated with the specimens immersed in a boron-rich mixture. As per the illustrative Fe-B phase diagram (see Figure 2), the temperature interval of boronizing ranged from 1123 K to 1273 K, with process durations spanning from 2 to 8 h. Once the surface hardening process was completed, the cylindrical-shaped container was then taken out of the furnace and placed at room temperature to cool. Subsequently, the specimen was extracted from the furnace and encased in acrylic resin using a Buehler SimpliMet 4000 (Lake Bluff, IL, USA) mounting press. This encasement was designed to facilitate the handling of the specimen during manual polishing, a decision based on the part’s shape and the intended analysis, which includes edge analysis as part of a thermochemical surface hardening process. The metallographic specimens underwent a thorough two-stage polishing procedure. Initially, the specimens were polished using a Buehler Micro-Cloth™ (Hong Kong, China), a soft, adaptable, commercial long pile artificial rayon fabric with magnetic mounting.

**Figure 3:**
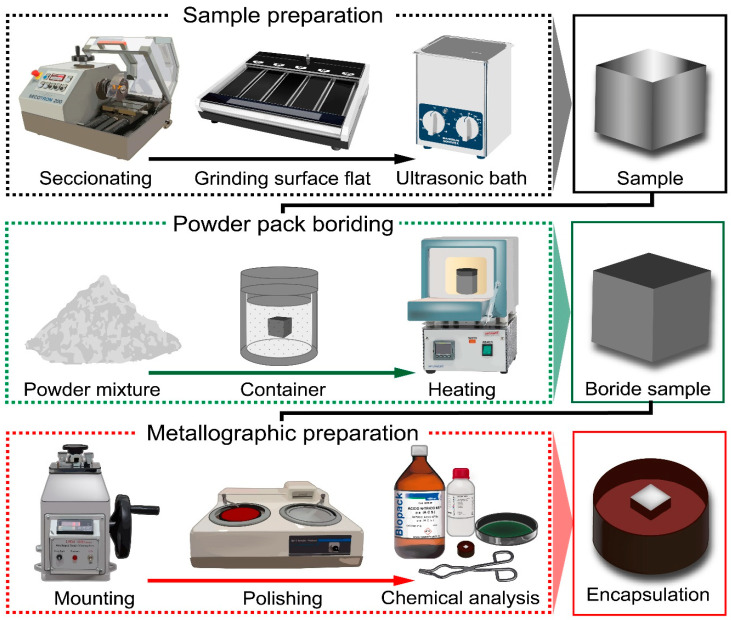
The flowchart diagram depicts the emission mechanism of the boron atom forming the diiron boronize phase in 35NiCrMo4 steel. The metallographic preparation consists of several sequential steps, including sectioning, roughing, ultrasonic bath treatment, thermochemical processing, mounting, polishing, chemical analysis, and examination using reflected light scanning microscopy (OM and SEM).

A cloth with Al_2_O_3_ abrasive (3 and 1 μm) was employed to eliminate any potential scratches resulting from the grinding process. Subsequently, the specimens were polished with a diamond suspension (0.25 and 0.05 × 10^−6^ m, Buehler) for 20 min at each size to achieve a specular surface essential for precise analysis. Following each step, the specimens underwent a 30 s ultrasonic acetone bath to ensure optimal cleanliness and quality. The specimen’ microstructure was analyzed using a 4% volume Nital immersion reagent. The thickness of the diiron boronize coatings was measured utilizing a VHX-7000 series 4K Optical Microscope (Higas-hi-Yodogawa-ku, Osaka, Japan) and a JEOL JSM-6510lV Scanning Electron Microscope (Akishima, Tokyo, Japan). Figure 3 illustrates the metallographic procedure, encompassing phase observations, grain boundaries, external agents, and distortion zones.

**Figure 4:**
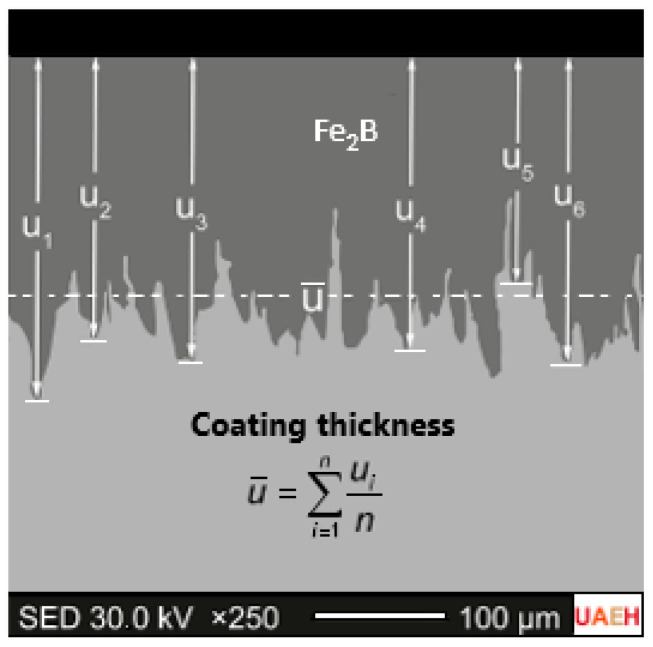
Sketch of the average thickness-measuring system of the diiron boronize coating.

The thickness of the Fe_2_B boronize coatings was determined using an Image-Pro Plus v.10.0 image analyzer (Rockville, MD, USA), a specialized software. An automated method was used for this purpose. The study on growth diffusion implied sixteen specimens, with each specimen being replicated once. To calculate the average thickness of the diiron boronize coating, 100 measurements were collected in each of the 32 targeted cross-sections. This method enabled the acquisition of precise and dependable data. Figure 4 provides a visual representation of the selected cross-sections.

## 3. Diffusion Model

**Figure 5:**
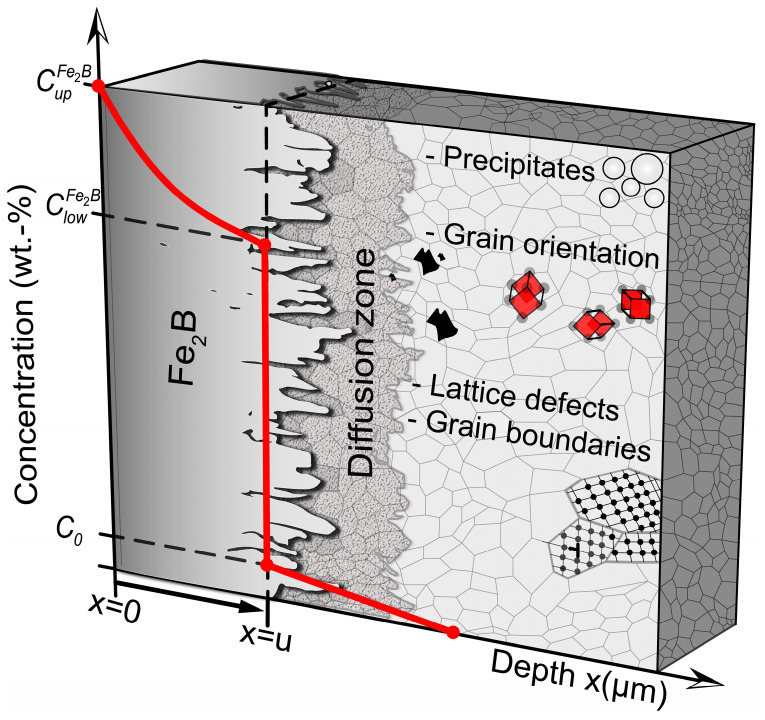
Figurative representation of the diiron boronize coating deposited on the surface of 35NiCrMo4 steel.

Understanding the diffusion of diiron boronize coatings’ growth on 35NiCrMo4 steel is essential for optimizing process parameters, thereby reducing manufacturing time and investment costs. Reducing the time required for processing to achieve the desired characteristics can result in significant energy savings and resource savings. A novel mathematical diffusion model was designed to analyze the diffusion of diiron boronize coatings (see Figure 5) settled on the surface of 35NiCrMo4 steel through solid boronizing. In Figure 5, a schematic representation of the boronized coating is illustrated. Overlaid on it is the concentration profile of the boronize coating with the respective boron limits at the boronize surface and diffusion interface (diiron boronize coating/substrate). In addition, the average value of the Fe_2_B coating thickness identified as u is mentioned.

**Figure 6:**
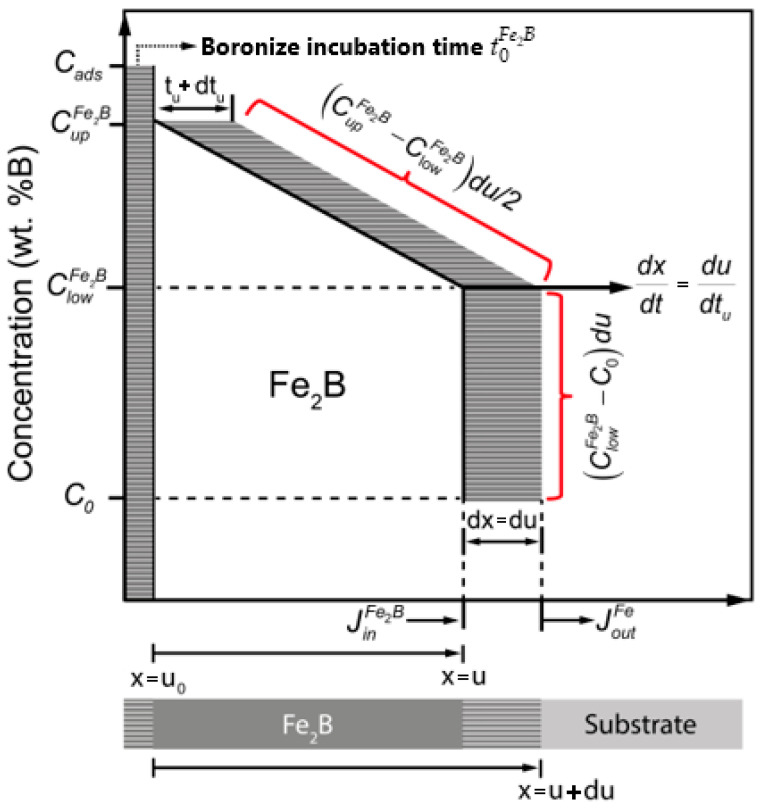
Graphical illustration of the B concentration graph through diiron boronize coating.

Figure 6 provides a figurative representation of the concentration graph within the Fe_2_B coating. Upon contact with the boronizing agent, boron atoms undergo thermodiffusion, permeating the Fe lattice to establish a saturated solid solution. After a boronize incubation time exceeding $t_{0}^{Fe_{2}B}$ (s), the formation of the Fe_2_B coating occurs, gradually becoming continuous and more densely packed with extended treatment. $C_{ads}$ represents the concentration of boron atoms (B) diffused at the Fe surface., $C_{up}^{Fe_{2}B}$ corresponds to the highest limit of B concentration in the diiron boronize coating (9 weight per cent of boron), $C_{low}^{Fe_{2}B}$ signifies the lowest limit of B concentration in the diiron boronize coating (8.83 weight per cent of boron), and x(t)=u (m) indicates the thickness of the diiron boronize coating. The concentration values at the surface and interface exhibit a very narrow concentration range, approximately one atomic per cent of boron, as documented by Brakman et al. [10]. The $C_{0}$ term, equivalent to 35 × 10^−4^ weight per cent of boron, signifies the threshold of boron solubility within the substrate, which can be disregarded. In Figure 6, the boron fluxes at the diiron boronize coating/Fe growth interface can also be seen, where $J_{in}^{Fe_{2}B}$ corresponds to the incoming boron flux just before the coating thickness boundary, i.e., at x=u, and $J_{out}^{Fe_{2}B}$ refers to the outgoing flux already in the substrate, i.e., at x=u+du.

The initial and boundary conditions of the mass transfer problem are given as follows [11]:

Time conditions:

(5) $$C_{Fe_{2}B}(x,t_{u}=0)=C_{0}=35\times 10^{-4}\;\mathrm{wt}.\%B.$$

Boundary conditions:

(6) $$C_{Fe_{2}B}(x=u_{0}\approx 0,t_{u}=t_{0}^{Fe_{2}B})=C_{up}^{Fe_{2}B}=9\;\mathrm{wt}.\%B,\;for\;C_{ads}≻8.83\;\mathrm{wt}.\%B,$$

(7) $$C_{Fe_{2}B}(x=u,t_{u}=t_{u})=C_{low}^{Fe_{2}B}=8.83\;\mathrm{wt}.\%B,\;for\;C_{ads}≺8.83\;\mathrm{wt}.\%B.$$

The parameter “$u_{0}$” represents the onset of a primary Fe_2_B (m) coating, terminating upon the apparition of the initial Fe boronizes after a specific durability characteristic of boronizes.

The B concentration graph is described by fundamental Fick’s second law as described below:

(8) $$\frac{\partial C_{Fe_{2}B}(x,t_{u})}{\partial t_{u}}=D_{Fe_{2}B}\frac{\partial^{2}C_{Fe_{2}B}(x,t_{u})}{\partial x^{2}}.$$

In cases where the boron diffusion coefficient is solely dependent on the boronizing temperature, Taylor’s series expansion can be used to derive the boron concentration profile within the Fe_2_B coating.

(9) $$C_{Fe_{2}B}(x,t_{u})=C_{Fe_{2}B}(a,t_{u})+{\left.\frac{\partial C_{Fe_{2}B}(x,t_{u})}{\partial x}\right|}_{x=a}(x-a)+{\left.\frac{\partial^{2}C_{Fe_{2}B}(x,t_{u})}{\partial x^{2}}\right|}_{x=a}\frac{{\left(x-a\right)}^{2}}{2!}.$$

For 0≤x≤u.

Taking a=u as the initial value and considering the boundary condition of Equation (7), the concentration profile of Equation (9), is expressed as follows:

(10) $$C_{Fe_{2}B}(x,t_{u})=C_{low}^{Fe_{2}B}+{\left.\frac{\partial C_{Fe_{2}B}(x,t_{u})}{\partial x}\right|}_{x=u}(x-u)+{\left.\frac{\partial^{2}C_{Fe_{2}B}(x,t_{u})}{\partial x^{2}}\right|}_{x=u}\frac{{\left(x-u\right)}^{2}}{2!}.$$

The B concentration graph depicted in Figure 6 ($C_{up}^{Fe_{2}B}$, $C_{low}^{Fe_{2}B}$, and $C_{0}$) remains constant concerning time and is expressed by the phase diagram presented in Figure 2. With consideration of these specific conditions, the mass balance at the diiron boronize coating/Fe substrate growth interface can be articulated as follows:

(11) $$\begin{array}{ll} \left(\frac{C_{up}^{Fe_{2}B}+C_{low}^{Fe_{2}B}-2C_{0}}{2}\right){\left.\left(\frac{dx}{dt_{u}}\right)\right|}_{x\;=\;u} & ={\left.J_{in}^{Fe_{2}B}(x)\right|}_{x=u}-{\left.J_{out}^{Fe}(x)\right|}_{x=u+du}\\ & =-D_{Fe_{2}B}{\left.\left(\frac{\partial C_{Fe_{2}B}(x,t_{u})}{\partial x}\right)\right|}_{x\;=\;u}-\left(-D_{Fe}{\left.\left(\frac{\partial C_{Fe}(x,t_{u})}{\partial x}\right)\right|}_{x\;=\;u+du}\right). \end{array}$$

$t_{u}(=t-t_{0}^{Fe_{2}B})$ (s) represents the effective phase formation time, which is expressed in terms of the treatment time t (s) and $t_{0}^{Fe_{2}B}$ (s). Additionally, $J_{in}^{Fe_{2}B}(x,t_{u})$ (atoms/m^2^·s) signifies the inward flux of B into the diiron boronize phase, while $J_{out}^{Fe}(x,t_{u})$ refers to the outward flux into the substrate (atoms/m^2^·s). $D_{Fe_{2}B}$ corresponds to the diffusion coefficient of B in the diiron boronize phase (m^2^/s). Understanding this parameter is key to gaining a comprehensive knowledge of the process. Last but certainly not least, $D_{Fe}$ takes the stage, referring to the diffusivity in the substrate (m^2^/s). The diffusivities in Equation (11) are independent of concentration. Consequently, direct approaches are available to tackle the issue of different boundary conditions. Notably, there is no B flux from the diiron boronize surface coating to the Fe, i.e., ${\left.J_{out}^{Fe}(x,t_{u})\right|}_{x=u+du}=0$, as depicted in Figure 6, which portrays the B concentration graph for this system. The thickness of the diiron boronize coating is denoted as “u” (μm). Upon rephrasing Equation (11), we obtain

(12) $$\left(\frac{C_{up}^{Fe_{2}B}+C_{low}^{Fe_{2}B}-2C_{0}}{2}\right){\left.\left(\frac{dx}{dt_{u}}\right)\right|}_{x\;=\;u}=-D_{Fe_{2}B}{\left.\left(\frac{\partial C_{Fe_{2}B}(x,t_{u})}{\partial x}\right)\right|}_{x\;=\;u}.$$

Multiplying both sides of Equation (12) by $\partial x/\partial t_{u}$, we have

(13) $${\left.\left(\frac{\partial C_{Fe_{2}B}(x,t_{u})}{\partial t_{u}}\right)\right|}_{x\;=\;u}=-\frac{1}{D_{Fe_{2}B}}\left(\frac{C_{up}^{Fe_{2}B}+C_{low}^{Fe_{2}B}-2C_{0}}{2}\right){\left(\frac{du}{dt_{u}}\right)}^{2}.$$

Combining Equation (13) and Equation (8), we obtain

(14) $${\left.\left(\frac{\partial^{2}C_{Fe_{2}B}(x,t_{u})}{\partial x^{2}}\right)\right|}_{x\;=\;u}=-\frac{1}{D_{Fe_{2}B}^{2}}\left(\frac{C_{up}^{Fe_{2}B}+C_{low}^{Fe_{2}B}-2C_{0}}{2}\right){\left(\frac{du}{dt_{u}}\right)}^{2}.$$

Equation (12) can also be expressed as

(15) $${\left.\left(\frac{\partial C_{Fe_{2}B}(x,t_{u})}{\partial x}\right)\right|}_{x\;=\;u}=-\frac{1}{D_{Fe_{2}B}}\left(\frac{C_{up}^{Fe_{2}B}+C_{low}^{Fe_{2}B}-2C_{0}}{2}\right)\left(\frac{du}{dt_{u}}\right).$$

Now, substituting Equations (14) and (15) in Equation (10), we arrive at

(16) $$C_{Fe_{2}B}(x,t_{u})=C_{low}^{Fe_{2}B}-\frac{1}{D_{Fe_{2}B}}\left(\frac{C_{up}^{Fe_{2}B}+C_{low}^{Fe_{2}B}-2C_{0}}{2}\right)\left(\frac{du}{dt_{u}}\right)(x-u)-\frac{1}{D_{Fe_{2}B}^{2}}\left(\frac{C_{up}^{Fe_{2}B}+C_{low}^{Fe_{2}B}-2C_{0}}{2}\right){\left(\frac{du}{dt_{u}}\right)}^{2}\frac{{\left(x-u\right)}^{2}}{2}.$$

Applying the condition to the boundary of Equation (6), we have

(17) $$C_{up}^{Fe_{2}B}=C_{low}^{Fe_{2}B}+\frac{1}{D_{Fe_{2}B}}\left(\frac{C_{up}^{Fe_{2}B}+C_{low}^{Fe_{2}B}-2C_{0}}{2}\right)\left(\frac{du}{dt_{u}}\right)u-\frac{1}{D_{Fe_{2}B}^{2}}\left(\frac{C_{up}^{Fe_{2}B}+C_{low}^{Fe_{2}B}-2C_{0}}{2}\right){\left(\frac{du}{dt_{u}}\right)}^{2}\frac{u^{2}}{2}.$$

We rewrite Equation (17) as follows:

(18) $$u\left(\frac{du}{dt_{u}}\right)-u^{2}\frac{1}{2D_{Fe_{2}B}}{\left(\frac{du}{dt_{u}}\right)}^{2}=2D_{Fe_{2}B}\left(\frac{C_{up}^{Fe_{2}B}-C_{low}^{Fe_{2}B}}{C_{up}^{Fe_{2}B}+C_{low}^{Fe_{2}B}-2C_{0}}\right).$$

whereas

(19) $$a=\frac{1}{2D_{Fe_{2}B}},$$

(20) $$b=2D_{Fe_{2}B}\left(\frac{C_{up}^{Fe_{2}B}-C_{low}^{Fe_{2}B}}{C_{up}^{Fe_{2}B}+C_{low}^{Fe_{2}B}-2C_{0}}\right).$$

Substituting Equations (19) and (20) in Equation (18), we obtain

(21) $$u\left(\frac{du}{dt_{u}}\right)-au^{2}{\left(\frac{du}{dt_{u}}\right)}^{2}=b.$$

The solution of Equation (21) is ex-pressed as

(22) $$u^{2}=-\frac{\sqrt{1-4ab}}{a}t_{u}+\frac{t_{u}}{a}+C_{1}.$$

The constant $C_{1}$ can be determined considering that when $x=u_{0}\approx 0$ for $t_{u}=t_{0}^{Fe_{2}B}$, Equation (22) is transformed into the following:

(23) $$u^{2}=\left(1-\sqrt{1-4ab}\right)\left(t_{u}-t_{0}^{Fe_{2}B}/a\right).$$

Now, substituting Equations (19) and (20) into Equation (23), we arrive at the following:

(24) $$u^{2}=2D_{Fe_{2}B}(t_{u}-t_{0}^{Fe_{2}B})\left(1-\sqrt{1-4\left(\frac{C_{up}^{Fe_{2}B}-C_{low}^{Fe_{2}B}}{C_{up}^{Fe_{2}B}+C_{low}^{Fe_{2}B}-2C_{0}}\right)}\right).$$

Rewriting Equation (24), we arrive at the parabolic growth law:

(25) $$u^{2}=4\varepsilon_{Fe_{2}B}^{2}D_{Fe_{2}B}(t_{u}-t_{0}^{Fe_{2}B}),$$

where

(26) $$\varepsilon_{Fe_{2}B}^{2}=\frac{1-\sqrt{1-4\left(\frac{C_{up}^{Fe_{2}B}-C_{low}^{Fe_{2}B}}{C_{up}^{Fe_{2}B}+C_{low}^{Fe_{2}B}-2C_{0}}\right)}}{2}=9.6\times 10^{-3}.$$

## 4. Results

### 4.1. Chemical Analysis (XRD, EDS)

**Figure 7:**
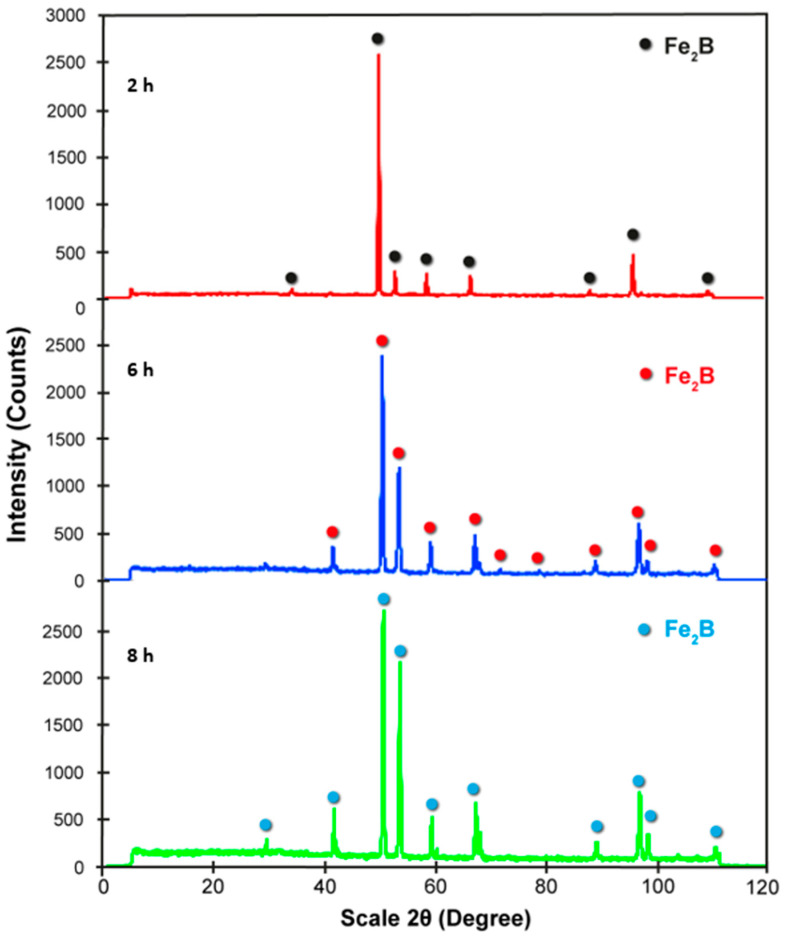
The X-ray image patterns obtained for the diiron boronize surfaces on 35NiCrMo4 steel at the highest temperature, 1273 K, for three different exposure times (2 h, 6 h, and 8 h) is presented. The results obtained were subjected to a thorough examination to identify the characteristic crystalline phase present on the substrate.

X-ray diffraction was conducted on boronize specimens to ascertain the composition of the boronize coating. The structural makeup of the diiron boronize coating, whether single-phase or multiphase, can significantly impact its tribological, mechanical, and adhesion properties. The XRD patterns were obtained using Rigaku Lab equipment (Chiyoda City, Tokyo, Japan), considering CuK_α_ radiation with a wavelength of 0.154 nm, a step magnitude of 0.01 degrees/second, and a 2θ range of 0 degrees–120 degrees. Figure 7 depicts the analysis of specimens subjected to temperatures of 1273 K for time periods of 2 h, 6 h, and 8 h. The analysis results indicate that the diiron boronize coating exhibits a single-phase structure, specifically Fe_2_B. In accordance with the ICDD reference number 00-036-1332, the main peaks observed at a diffraction angle of 52 degrees correspond to the three highest intensity values for the Fe_2_B phase. These peaks are evident in the X-ray image patterns of all analyzed specimens, reaffirming the presence of the Fe_2_B phase in the coatings. 

**Figure 8:**
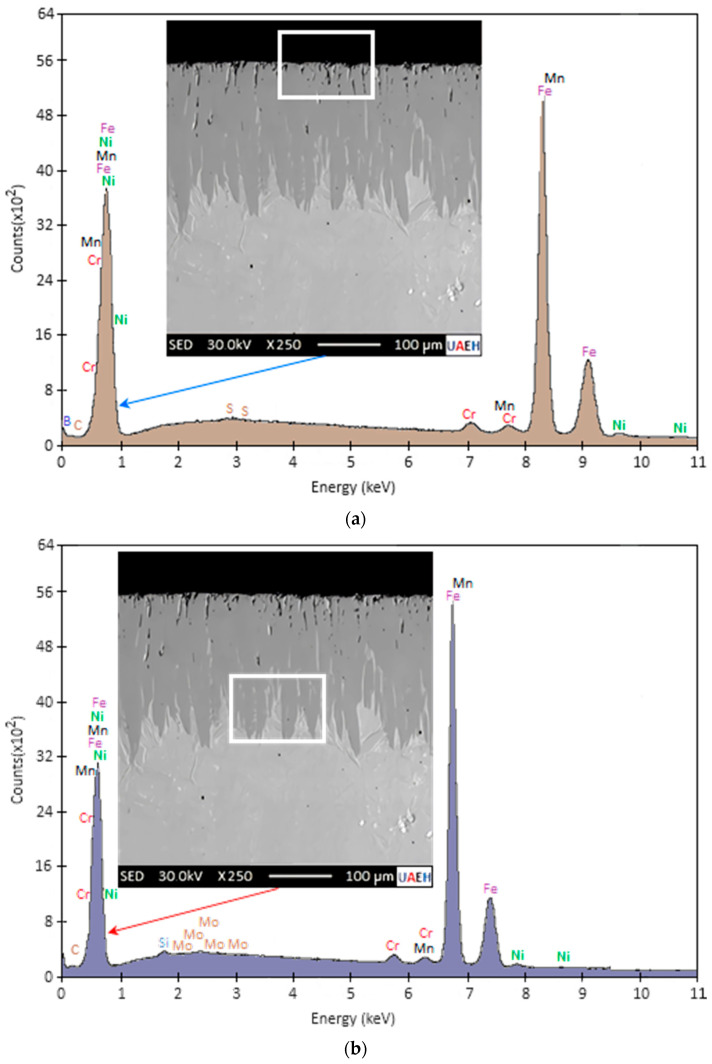
A chemical analysis of electron dispersive spectroscopy (EDS) was performed on the cross-sectional area of 35NiCrMo4 steel subjected to the thermochemical surface hardening process at the highest temperature of 1273 K for an exposure time of 8 h. Two different regions were analyzed, namely (**a**) on the specimen surface and (**b**) near the diiron boronize coating/Fe growth interface.

The vertical cross-section of a specimen hardened for the highest temperature of 1273 K and an exposure time of 8 h was examined by Scanning Electron Microscopy (SEM), and the qualitative results of the area energy dispersive X-ray spectroscopy (EDS) analysis are presented in Figure 8. The EDS analyses aimed to identify the possible presence of elements such as iron (Fe) and boron (B) in the diiron boronize coating and determine their chemical concentrations. The elemental analysis was carried out in two distinct regions: on the surface of the diiron boronize coating (see Figure 8a) and at the growth interface (diiron boronize coating/Fe) (see Figure 8b).

After evaluation of the results obtained in the regions in areas 1 (as shown in Figure 1) and 2 (as shown in Figure 2) of the hardened specimen, the presence of the chemical element B (boron) could be determined. It is well established in several previous studies that the boronize coating deposited by the thermochemical boronizing treatment of unalloyed or low-alloyed iron-based steels can contain the diiron boronize or diiron boronize + monoboronize phase. According to the illustration of the iron–boron phase graph mentioned in Figure 2, the diiron boronize and monoboronize phases contain an approximate value of 9% by weight of B and 16.23% by weight of B, respectively [12]. According to the results obtained with EDS analysis, the diiron boronize coating contained approximately 8.25% by weight of element B, closely matching the theoretical weight percentage of element B in the diiron boronize phase. It is important to note that the EDS method can require high sensitivity to accurately detect chemical elements with low atomic numbers, particularly those below the value of eight unless the detector employed is made of silicon or lithium. Consequently, it was determined that the boriding processes formed a single-phase diiron boronize coating on the surface of 35NiCrMo4 steel. The analysis revealed the insolubility of chemical elements such as Si, C, and Mo, while elements such as Cr, Mn, and Ni were present in the coating. In agreement with the findings reported in the literature, it was observed that hardened coatings could dissolve elements such as Cr, Ni, and Mn. In addition, the change in the concentration of element B from the surface to the matrix (1→2) indicated a decrease in the amount of element B towards the matrix.

### 4.2. Study of the Microstructure of the Diiron Boronize Coating (Optical Microscopy)

**Figure 9:**
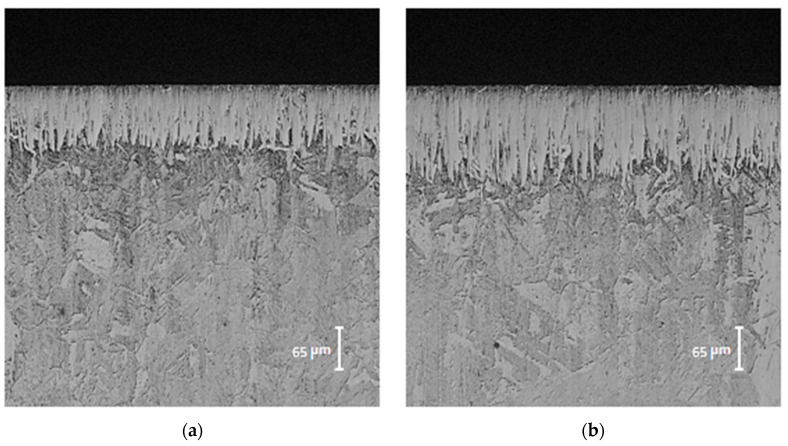

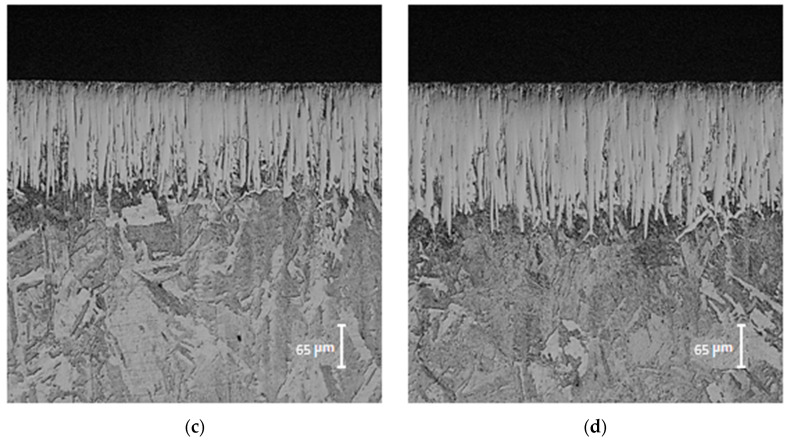
Optical micrographs of diiron boronize coatings developed on the surface of 35NiCrMo4 steel, obtained at a maximum temperature of 1273 K, with varying exposure durations: (**a**) 2 h, (**b**) 4 h, (**c**) 6 h, and (**d**) 8 h.

The illustrations in Figure 9 show vertical areas of the OM-hardened specimens. These images reveal an internal structure composed of two central regions: a diiron boronize coating and an underlying matrix region (substrate). By carefully examining the diiron boronize coatings of all the treatment-hardened specimens, it was observed that they exhibited a continuous presence on the material’s surface and showed uniform and consistent thicknesses. 

The images in Figure 9 show a “sawtooth” morphology, characteristic in low-alloy steels due to the anisotropic diffusion process of boron in the metal matrix, which causes an inhomogeneous growth of the boronize phase. This behavior of Fe_2_B iron boronizes is related to its tetragonal crystal structure characterized by its low symmetry, favoring its preferential growth to the (002) plane, which is more compact and has the lowest surface energy [13]. Using Electron Back Scattered Electron Diffraction Spectroscopy (EBSD) analysis, a high concentration of compressive stresses was observed between the decreasing grains of Fe_2_B iron boronize; these stresses limit their lateral growth, forcing them to a unidirectional growth inside the matrix [13]. 

**Figure 10:**
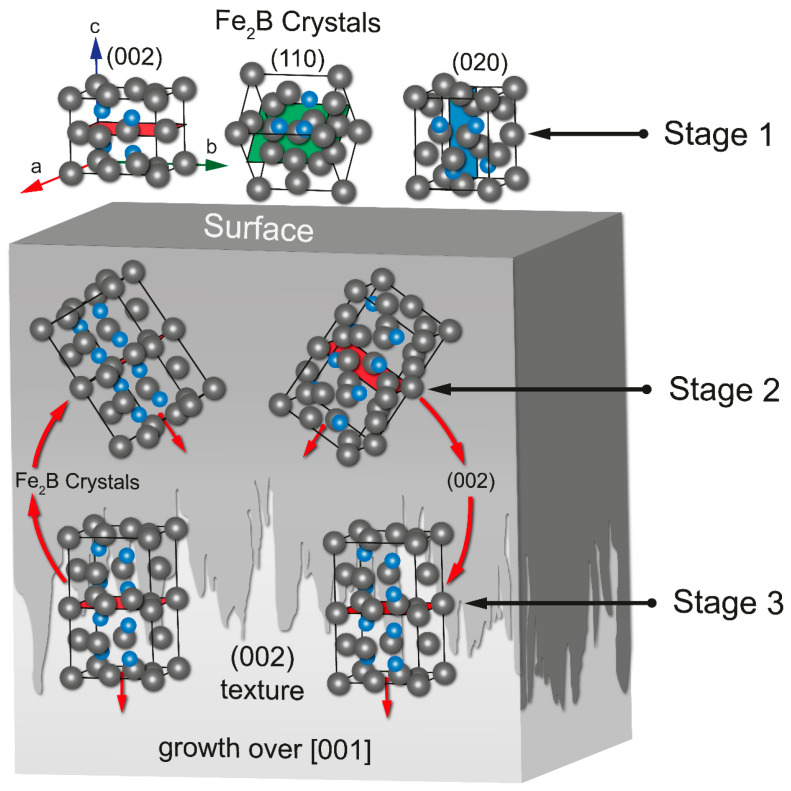
The growth stages of iron boronizes can be categorized as follows: Stage 1 involves the random growth of crystals on the surface of the base material. Stage 2 is characterized by the internal growth of diiron boronize crystals within the base material. Stage 3 entails an increase in the thickness of the diiron boronize coating, along with the development of a defined texture in the crystallographic plane (002). The grey spheres illustrate iron atoms, while the blue spheres refer to boron atoms.

The optical micrographs illustrated in Figure 9 demonstrate the evolution of diiron boronize thickness as the experimental variables are increased. This change is attributable to the thermally activated nature of the treatment; specifically, as the exposure time is extended, a greater quantity of boron atoms (B) diffuses from the surface into the metal matrix, contributing to the increased thickness of the diiron boronize coating. Figure 9 further indicates that the hard coating exhibits a characteristic sawtooth morphology and is predominantly single-phase [14]. Furthermore, the atomic density of boron (B) within the tetragonal crystal lattice associated with the diiron boronize phase shows a preference for the crystallographic direction [001] (as detailed in Figure 10). Figure 10 provides a visual representation of the mechanism underlying the formation of iron boronizes across three distinct stages.

### 4.3. Microstructural Characterization of Boronize Coating (Scanning Electron Microscopy)

**Figure 11:**
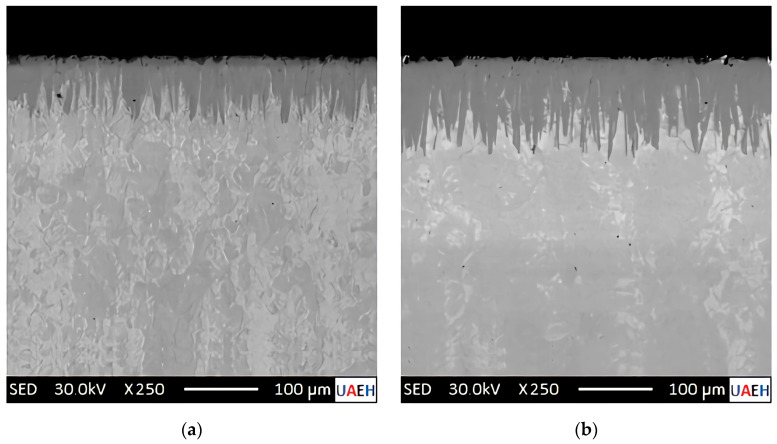

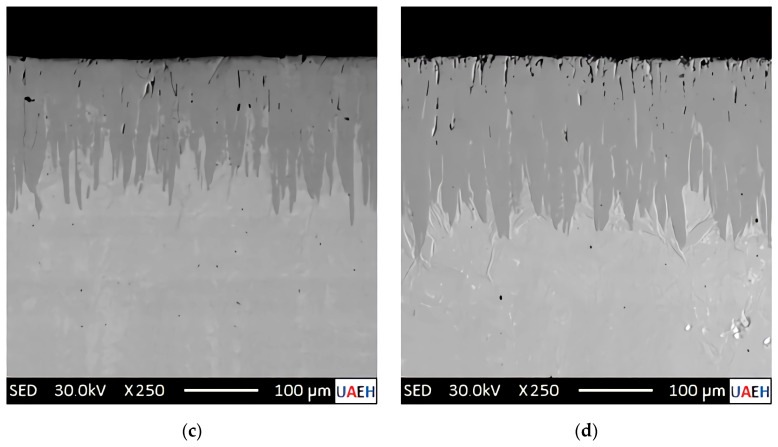
Photomicrographs obtained through Scanning Electron Microscopy (SEM) illustrate the cross-sectional areas of boronized coatings formed on the surface of 35NiCrMo4 steel. The steel was treated at an elevated temperature of 1273 K for varying exposure durations: (**a**) 2 h, (**b**) 4 h, (**c**) 6 h, and (**d**) 8 h.

Figure 11 shows SEM micrographs of the cross sections of the boronized specimens using various processing parameters.

The images in Figure 11 are secondary electron micrographs obtained by SEM at an accelerating voltage of 30 kV. Upon examination of microstructure photographs from transverse sections of the hardened specimens, a noticeable contrast discrepancy is observed in the re-coated region where monocoating (Fe_2_B) and substrate (Fe) formation occur. This distinction is attributed to the Fe_2_B phase, which exhibits an atomic boron concentration of 9 wt.% B within a solid solution and a density of $\rho_{Fe_{2}B}=$ 7430 kg∙m^−3^, as detailed in prior research [9,11]. Following a comprehensive analysis of the microstructure photographs from all specimens within this study, and after careful consideration of the observed phenomenon, it can be concluded that the diiron boronize phase is solely responsible for the formation of the boronized coatings. This conclusion is supported by the X-ray diffraction (XRD) analysis presented in Section 4.1.

### 4.4. Microhardness

**Figure 12:**
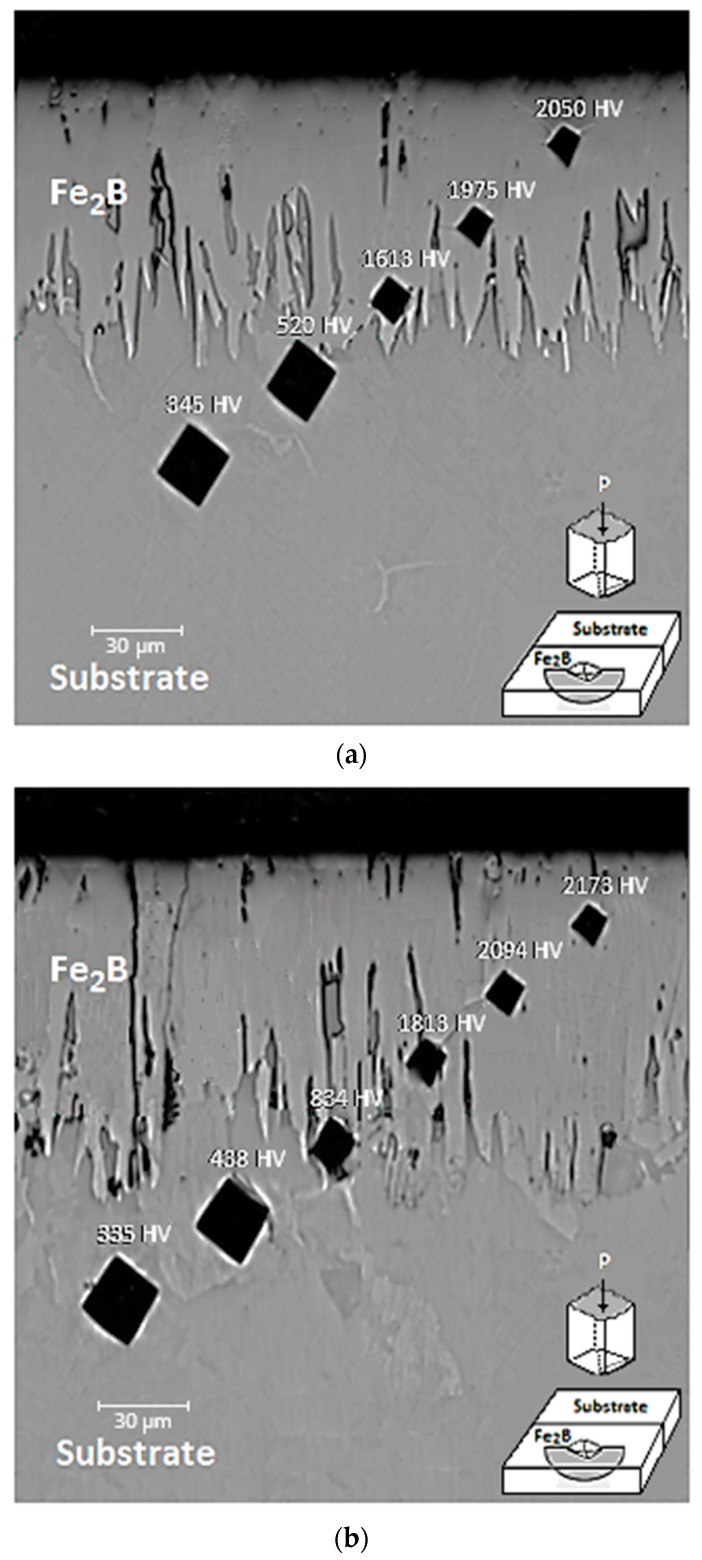
Photomicrographs obtained through optical microscopy (OM) illustrate the diiron boronize coatings’ Vickers hardness profiles for two hardened specimens of 35NiCrMo4 steel. The specimens were treated for two temperatures: (**a**) 2 h at 1273 K and (**b**) 4 h at 1223 K, considering a load of 98.1 × 10^2^ N.

The medium microhardness measurements of the boronized coating and the matrix regions were evaluated by Vickers microhardness measurements performed on the cross sections of the specimens after the thermochemical surface hardening process (refer to Figure 12). The impact of treatment parameters on the hardness values was assessed. The surface coatings of the specimens exhibit a single-phase structure. As per the existing literature, the hardness of a single-phase boronize coating consisting solely of the Fe_2_B phase can reach levels of approximately 1800–2000 HV [15]. Analysis of the measurement results indicated that the boronized materials exhibited a notably high surface hardness, attributable to the development of the boronize coating on their surface.

The average surface hardness values of specimens Figure 12a,b were determined to be 2050 HV and 2173 HV, correspondingly. It can be seen that the levels of hardness of the coatings increase with extended boronizing time and higher temperatures. When the hardness measurements in the matrix region are examined, the average hardness value is 340 HV. This indicates that the thermochemical surface hardening process considerably improves the surface hardness of the 35NiCrMo4 material by approximately 6.2 times. Previous research (references [15,16]) has established that the hardness values of diiron boronize coatings are primarily influenced by several critical parameters. These include thermal residual stresses, temperature, treatment duration, the chemical composition of the metallic matrix, and the grade of anisotropy present in the boronize coating.

### 4.5. Estimation of the Young’s Modulus of Diiron Boronized Coatings by Means of Nanoindentation

**Figure 13:**
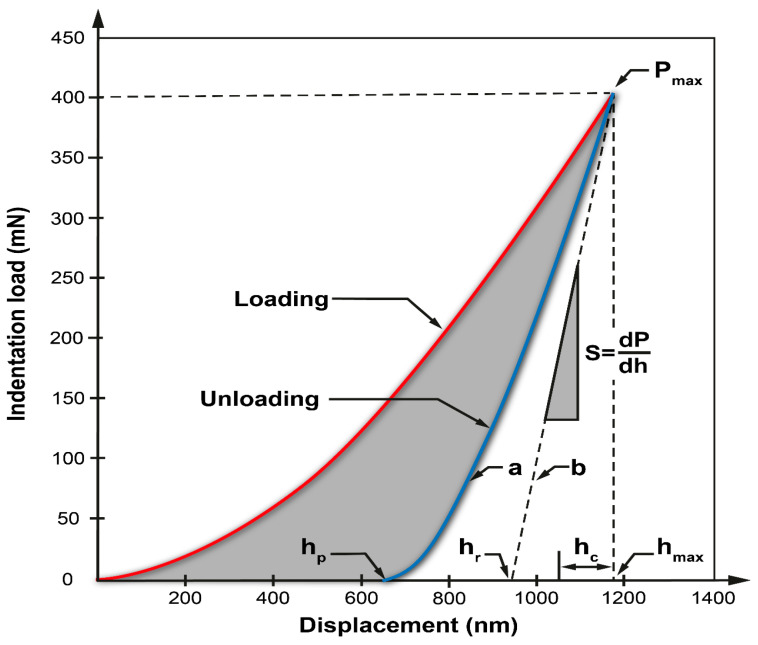
Diagram illustrating standard load vs. indenter displacement data in a nanoindentation experimental setup.

The Young’s modulus was estimated using the instrumentation indentation technique using a Table Top Nanoindentation Tester (TTX-NHT, CSM Instruments) (Needham, MA, USA) according to the methodology proposed by Oliver and Pharr and employing a Berkovich indenter [17]. In the field of nanoindentation, the methodology developed by Oliver and Pharr quantifies the depth of penetration of a diamond indenter in conjunction with the applied load. The resulting load–displacement curve generally displays a behavior characterized by an elastic–plastic loading phase, followed by an elastic unloading phase, as exemplified in Figure 13. Next, the elastic contact elastic equations are used along with the unloading data to determine the Young’s modulus and surface hardness of two specimens treated at 1273 K for a 2 h exposure duration and at the temperature of 1223 K for a 4 h exposure duration.

A series of five indentations were performed on the diiron boronize phase surface. The measurements were conducted with a critical load of 50 mN at a loading and unloading rate of 100 mN/min. As a result of these calculations, the load–displacement function curves, the indentation hardness, and the Young’s modulus of the indentation were calculated. Different types of indenters are used in nano hardness. However, the Berkovich indenter with a triangular pyramidal geometry with an angle of 65.3° is usually used in most studies. The geometry of the Berkovich indenter is meticulously designed to establish a consistent relationship between projected area and depth of penetration, similar to that of the Vickers indenter. This design ensures the reliability and comparability of measurements in nanoindentation experiments, facilitating accurate assessments of material hardness and mechanical properties. However, it has the advantage of being manufactured with higher pre-precision at the nanometer scale as it has only three faces. The Oliver and Parrs method allows us to analyze the results of the nano hardness test by evaluating three critical parameters of the load–displacement curve: the maximum load $F_{max}$, the highest displacement $h_{max}$, and the unloading rigidity S=dP/dh. A numerical fit to the power-law function was used to analyze the unloading curve, and the data were considered to be in the range of 98–40% of the maximum load [18]:

(27) $$F=F_{max}{\left(\frac{h-h_{p}}{h_{max}-h_{p}}\right)}^{m}.$$

The power m is a parameter of the numerical setting, and $h_{p}$ is the deformed displacement. During the unloading process, mechanical phenomena such as creep or viscoelasticity occur and affect the results, so the value of $h_{max}$ cannot be used directly to obtain information. Similarly, the complex behavior of the contact mechanics at the lower end of the unloading characteristic curve makes it difficult to use the value of $h_{p}$. For this reason, the contact stiffness S is used as a reference parameter. The contact stiffness corresponds to the inclination of the tangent line to the unloading curve and, for the analysis, is usually evaluated at the point of maximum displacement and can be calculated as follows:

(28) $$S=m\cdot F_{max}{\left(h_{max}-h_{p}\right)}^{-1}.$$

Crossing of the tangent line of the unloading curve with the displacement axis $h_{r}$ (see Figure 13) is determined as follows:

(29) $$h_{r}=h_{max}-\frac{F_{max}}{S}.$$

The depth of contact $h_{c}$ of the penetrator with the specimen tested at load $F_{max}$ is determined by the equation below:

(30) $$h_{c}=h_{max}-\varepsilon (h_{max}-h_{r}).$$

The constant ε=0.75 depends on the shape of the Berkovich indenter. The projected contact area is calculated as follows:

(31) $$A_{p}=24.5h_{c}^{2}.$$

Knowing these data, the indentation hardness $H_{IT}$ can be evaluated with the following equation:

(32) $$H_{IT}=\frac{F_{max}}{A_{p}}.$$

The conversion for the Vickers $VH_{IT}$ scale from a measurement with a Berkovich indenter is usually expressed as

(33) $$HV_{IT}=\frac{HV_{IT}[MPa]}{10.800}.$$

The reduced elastic modulus $E_{r}$ is computed from the equation below.

(34) $$E_{r}=\frac{\sqrt{\pi }S}{2\beta \sqrt{A_{p}}}.$$

β=1.034 is a geometrical correction factor for the triangular indenter. To obtain the plane strain modulus $E^{*}$, the following expression is used.

(35) $$E^{*}=\frac{1}{\frac{1}{E_{r}}-\frac{1-\nu_{i}}{E_{i}}}.$$

$E_{i}=1141$ GPa corresponds to the diamond’s elastic modulus and Poisson’s ratio $\nu_{i}=0.07$. To calculate the indentation Young’s modulus from $E^{*}$, a Poisson’s ratio value estimated for the Fe_2_B $\nu_{s}=0.25$ phase was used [12,18].

(36) $$E_{IT}=E^{*}(1-\nu_{s}^{2}).$$

**Figure 14:**
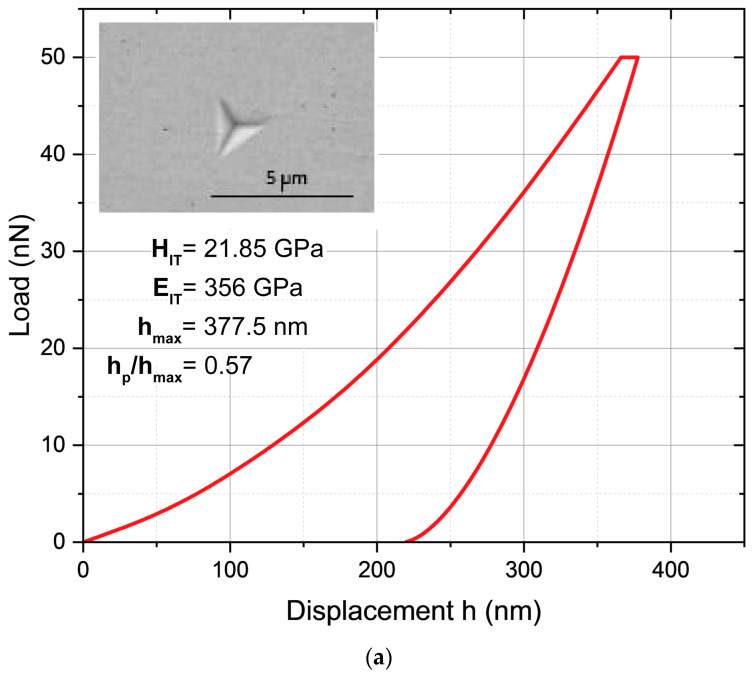

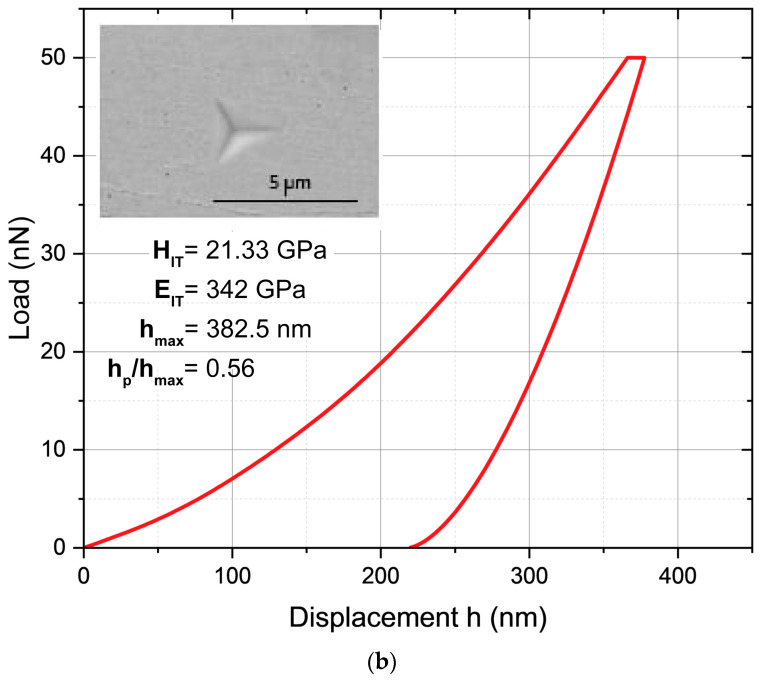
SEM images of 35NiCrMo4 steel after application of the boronizing treatment with selected visible indentations and surface nanoindentation results: (**a**) 2 h at 1273 K and (**b**) 4 h at 1223 K.

Figure 14 presents Scanning Electron Microscopy (SEM) images of 35NiCrMo4 steel, illustrating observable Berkovich penetrations conducted at a temperature of 1273 K for a duration of 2 h (Figure 14a). The measured hardness and Young’s modulus at the indentation were 21.85 GPa and 356 GPa, respectively. In a subsequent indentation performed on the hard surface at a temperature of 1223 K over a period of 4 h, a slightly lower hardness ($H_{IT}$) of 21.33 GPa and a reduced Young’s modulus ($E_{IT}$) of 342 GPa were recorded (Figure 14b). The results are consistent with the hardness and surface Young’s modulus values for the diiron boronized phase as given in the relevant literature [12].

### 4.6. Rockwell-C Indentation Coating Adhesion Tests

**Figure 15:**
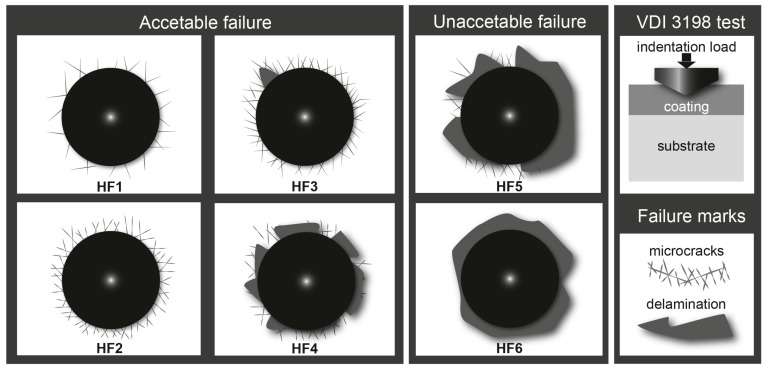
Different configurations of cohesive strength quality are utilized to determine the cohesion of the diiron boronized coatings adhered to the metallic alloy, as determined by the Daimler-Benz Rockwell-C indentation test.

Tribological testing is essential for assessing the adhesion of diiron boronized coatings to their substrates. Figure 15 illustrates the various configurations of adhesion quality (HF1–HF6) utilized in the evaluation of damage to the diiron boronized coating. Generally, the adhesion of the diiron boronized coating is deemed adequate for configurations HF1 through HF4, whereas configurations HF5 and HF6 are classified as having insufficient adhesion. The craters produced during these tests were matched against the 3198 standard, which is endorsed by the Association of German Engineers (in German, it is called Verein Deutscher Ingenieure Normen) [19].

**Figure 16:**
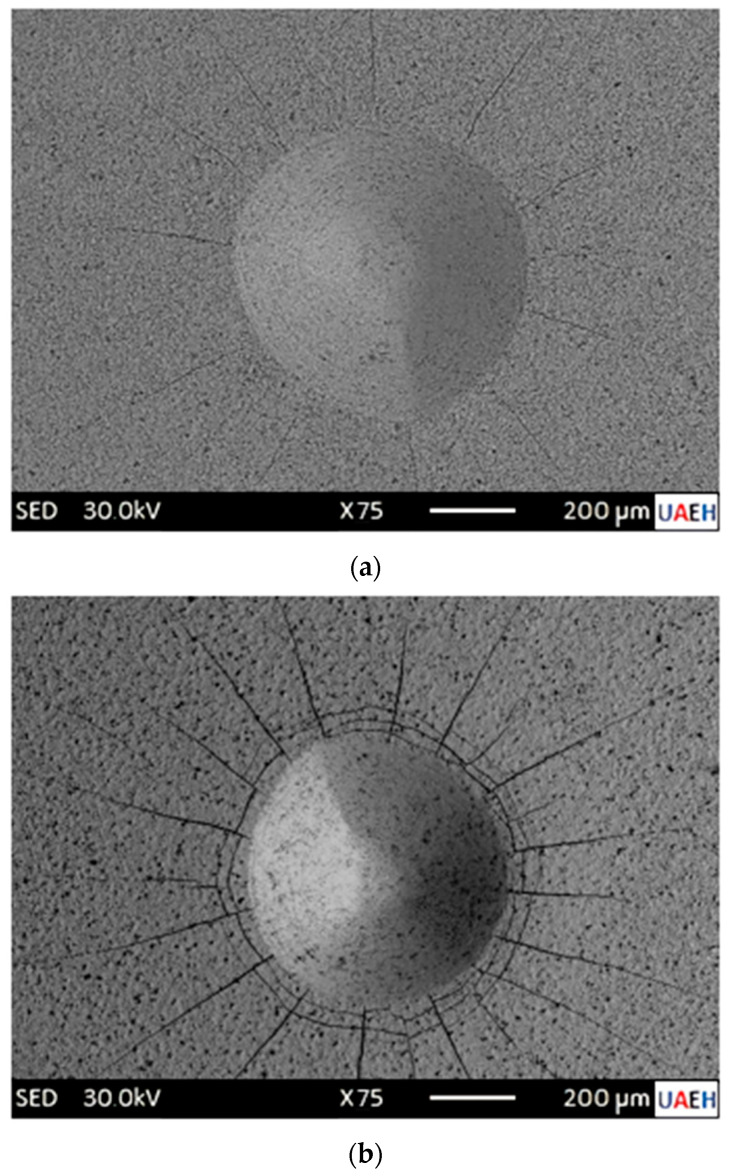
The Scanning Electron Microscopy (SEM) micrographs depict indentations resulting from the VDI adhesion inspection performed on 35NiCrMo4 steel surfaces subjected to boronizing for (**a**) 2 h at 1123 K and (**b**) 8 h at 1273 K.

The cohesive tests performed using the Daimler-Benz Rockwell-C method on 35NiCrMo4 steel surfaces, hardened at temperatures of 1123 K and 1273 K for a duration of 8 h, produced significant results. The Scanning Electron Microscope (SEM) images presented in Figure 16 effectively illustrate the impact of the indenter on the treated specimens. In Figure 16a, the specimen treated at 1123 K for 8 h exhibited no delamination on the crater surface, indicating the presence of radial cracks without delamination, thereby satisfying the HF1 category criteria. In contrast, Figure 16b demonstrates delamination at the periphery of the crater and radial cracks on the boronized surface of the specimen treated at 1273 K for 8 h, which qualifies it under the HF4 category. These findings suggest that the diiron boronized coating maintains an acceptable level of cohesive quality in relation to the substrate.

According to reference [19], the interfacial cohesion of the diiron boronized coating is significantly influenced by the phase composition, particularly in relation to the diiron boronized coating thickness ratio, and is also dependent on the treatment temperature. In a similar study, Kayali and Kara [20] reported comparable findings. The diiron boronized coating that was applied to the surface of Hardox-450 steel underwent assessment using the Daimler-Benz Rockwell-C cohesion test. The results indicated that diiron boronized coatings produced at 1123 K for a duration of 2 h exhibited superior interfacial cohesion, categorizing them within the HF1 classification. In contrast, coatings generated at 1223 K for 6 h were classified as HF5, which is considered unacceptable.

### 4.7. Coefficient of Friction (COF)

**Figure 17:**
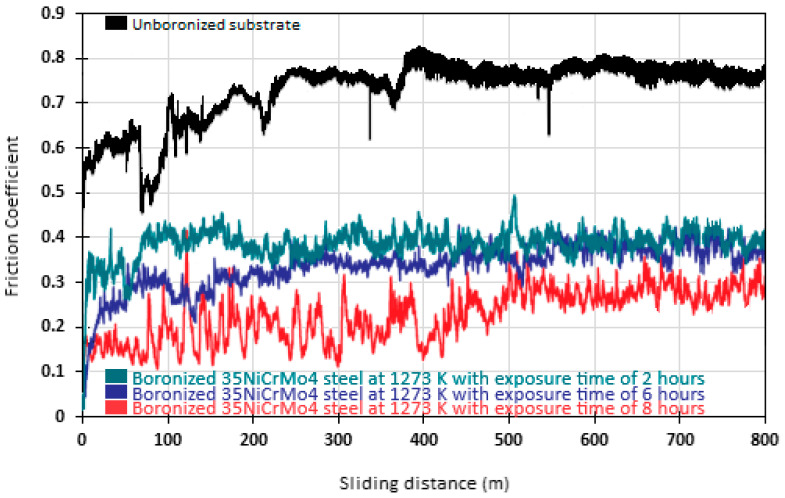
Representation of the COF with respect to the sliding distance L for wear tests on 35NiCrMo4 steel, showing distinct behaviors depending on the testing duration. Measurements were taken in the initial state (substrate) and after 2 h, 6 h, and 8 h of treatment at a temperature of 1273 K.

Figure 17 illustrates the relationship between the COF and the sliding distance on both untreated and diiron boronized specimens at 1273 K for durations of 2, 6, and 8 h. The tribological characteristics of the four surfaces tested under dry conditions were assessed by subjecting the surfaces to a diamond indenter. The findings reveal a notable contrast in the COF between the three boronized surfaces and the substrate, attributable to the development of a durable diiron boronized coatings on the surface of the specimens. The average COF of the boronized surfaces at a temperature of 1273 K exhibited variations, with values of 0.283 for 8 h, 0.371 for 6 h, and 0.397 for 2 h. In contrast, the untreated substrate demonstrated a notably higher value at approximately 0.756. The study undertook a comparative analysis alongside previously published studies, demonstrating consistency with data communicated by other researchers irrespective of the treatment conditions applied. The COF values derived from this study for 35NiCrMo4 steel exhibit close alignment with the existing literature. For example, Selçuk et al. [21] documented variable COF values varying from 0.36 to 0.62 for carbon steels. Additionally, Venkataraman and Sundararajan [22] examined the wear resistance of carbon steel with a thermochemical surface hardening process, identifying a COF value that varied between 0.3 and 0.5 depending on the sliding velocity.

### 4.8. Evaluating Boron Activation Energy for 35NiCrMo4 Steel

**Figure 18:**
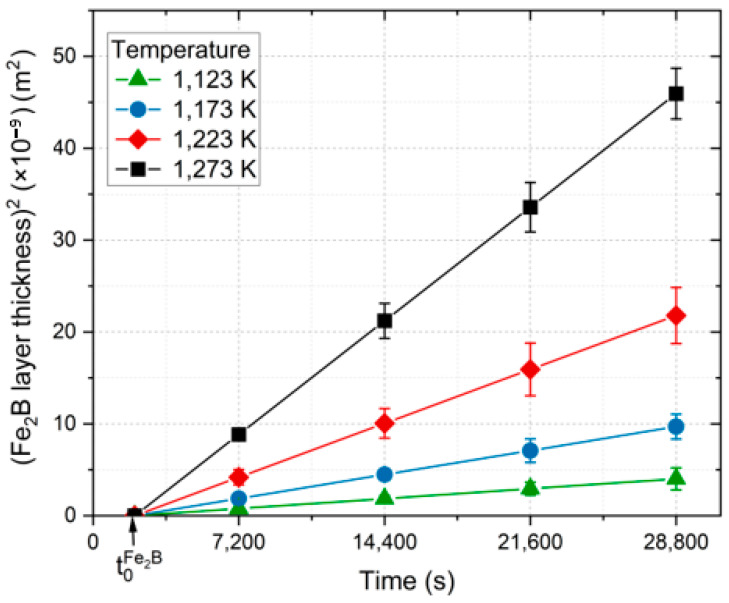
Graph of the different squared coating thicknesses obtained for varying treatment temperatures formed on the surface of the metal alloy (35NiCrMo4) versus the phase formation time.

The calculation of boron diffusion coefficients necessitated the determination of the characteristic law of growth of iron boronizes, as outlined in Equations (25) and (26) in Table 1. The slopes of the graphs depict the correspondence between the squared coating thickness and the formation time, as illustrated in Figure 18. It is noteworthy that the characteristic incubation periods exhibited similar values across the selected temperature range.

**Figure 19:**
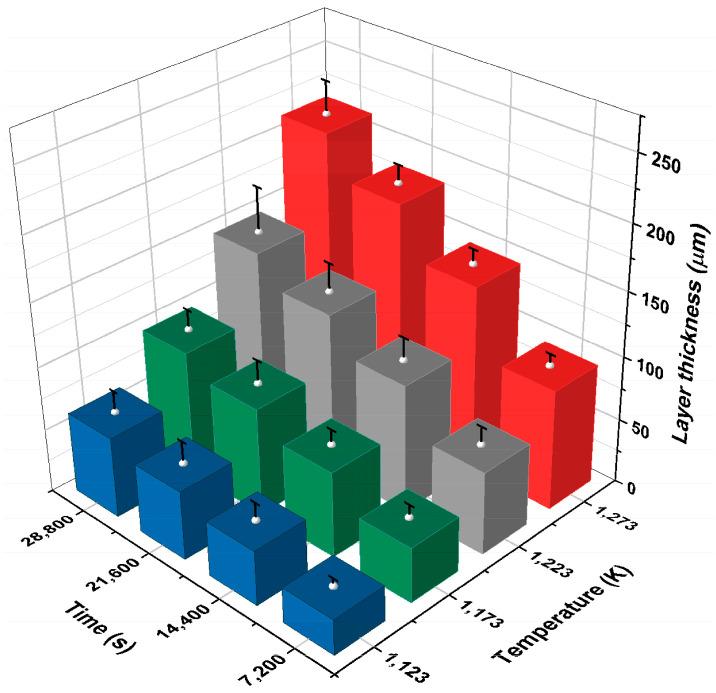
The value of the diiron boronized thickness as a dependence of processing time and temperature.

The diiron boronized coatings deposited on the surface of 35NiCrMo4 metal alloy varied from 27.806 to 214.35 μm due to different treatment times and temperatures. Figure 19 shows a 3D plot of the correspondence between the thickness of the diiron boronized coating, temperature, and processing time. The thickness increases in magnitude with respect to the two experimental parameters [23].

**Figure 20:**
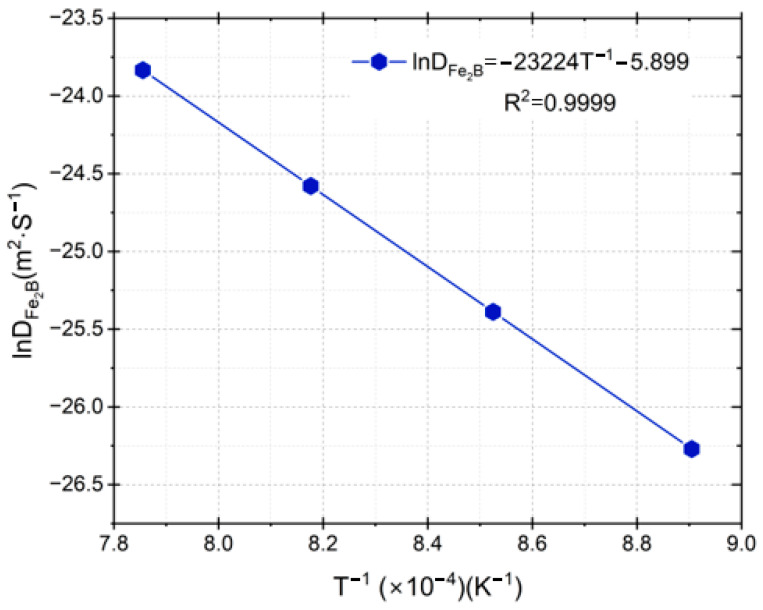
In the graph between the diffusivity’s natural logarithm and the temperature inverse, the slope obtained is related to the activation energy.

The diffusion coefficients ($D_{Fe_{2}B}$) from Table 1 were fitted to the classical Arrhenius relation [20], as illustrated in Figure 20.

Equation (27) represents the diffusivity of boron in the diiron boronized phase, which was obtained by the least square method from the experimental data in Figure 18 as follows:

(37) $$D_{Fe_{2}B}=3.7355\times 10^{-3}exp\left(-193\;\mathrm{kJ}\cdot {\mathrm{mol}}^{-1}/RT\right).$$

The parameter *R* (=8.314 J/mol·K) is a classical constant that is associated with ideal gases, while *T* is indicative of the temperature. Figure 18 illustrates the relationship between $lnD_{Fe_{2}B}$ and the inverse of temperature; the resulting slope enables us to establish its correlation with the lowest value of the potential energy barrier of boron in diiron boronized coating for 35NiCrMo4 steel, denoted as 193 kJ∙mol^−1^. Additionally, Table 2 provides a comparative analysis of the boron activation energy value derived from our current study with those documented for various boronizing steels [20,23,24,25,26,27,28].

**Figure 21:**
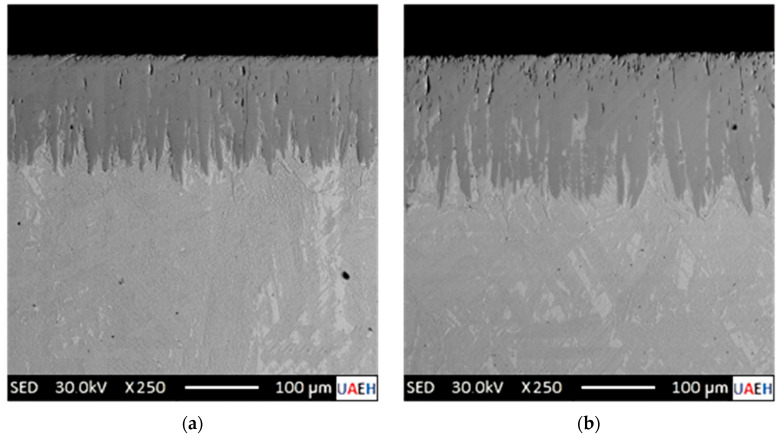
Photomicrographs obtained through Scanning Electron Microscopy (SEM) illustrate the cross-sectional areas of boronized coatings formed on the surface of 35NiCrMo4 steel. The steel was treated at an elevated temperature of 1323 K for varying exposure durations: (**a**) 1.5 h and (**b**) 2.5 h.

The SEM images in Figure 21 show the transversal scans of the specimens subjected to two different boronizing setups. Coating thickness data obtained at 1323 K for 1.5 h and 2.5 h are tabulated in Table 3, with calculated values derived using Equation (25). Remarkably, the predicted diiron boronized coating thicknesses closely align with the collected findings.

**Figure 22:**
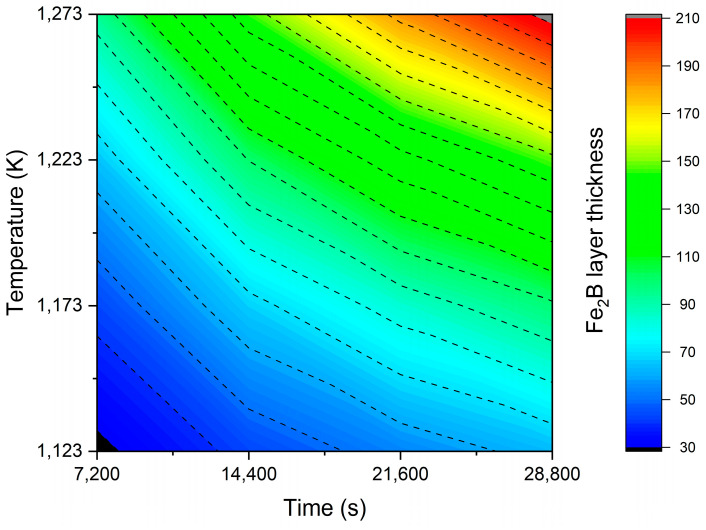
Contour plot of diiron boronized coating thickness as a function of the experimental parameters of the process.

Figure 22 illustrates a thickness iso-diagram depicting temperature in terms of exposure time ($T=-Q/Rln(u^{2}/4\varepsilon_{Fe_{2}B}^{2}D_{0}(t_{u}-t_{0}^{Fe_{2}B}))$). This contour plot is a valuable tool for determining the optimal Fe_2_B coating thickness for potential industrial applications of boronized 35NiCrMo4 steel.

### 4.9. ANOVA Analysis

Another important tool for analyzing the thermochemical surface hardening process is the analysis of variance (ANOVA), also referred to as factor analysis, which was introduced by Fisher in 1930. It serves as an essential statistical tool for evaluating the effects of one or more parameters —each having two or more levels—on the mean of a continuing variable [29]. It is the preferred statistical test for comparing the means of two or more groups. Additionally, this technique can be extended to explore factors’ potential effects on a variable’s variance. The null hypothesis for various forms of ANOVA posits that the mean of the variable being studied is the same across the other groups, as opposed to the alternative hypothesis, which suggests that at least two means differ significantly. Analysis of variance (ANOVA) facilitates the comparison of multiple means by examining variances. In our study, we focused on two factors, namely time (*t*) (7200, 14,400, 21,600, 28,800 s) and treatment temperature (*T*) (1123, 1173, 1223, and 1273 K), to assess their influence on the thickness growth of diiron boronized coating created on the surface of 35NiCrMo4 steel. As both factors have four levels, we employed the default 4 × 4 factorial design in Minitab Statistical Software Version 21.1.0 (State College, PA, USA) for the experiment. It is clear that the null hypothesis H_0_ posits that there is no significant influence of the factors of processing time and temperature, as well as their interaction (*t* × *T*), on the thickness of the diiron boronized coating. Conversely, the alternative hypothesis H_1_ suggests that at least one of the factors (treatment time or temperature) or their interaction (*t* × *T*) significantly impacts the Fe_2_B coating thickness.

Table 4 displays the outcomes of the factorial experiment evaluation. Within this context, the degrees of freedom denote the number of values capable of varying in the analysis. The sum of squares serves as an indicator of the total variability within the data. Principal squares represent the sum of squares divided by the corresponding degrees of freedom. The F-value denotes the ratio of between-group variability to within-group variability. Additionally, the contribution signifies the percentage of total variability for each factor, while the *p*-value reflects the probability that the observed differences are attributed to chance. 

**Figure 23:**
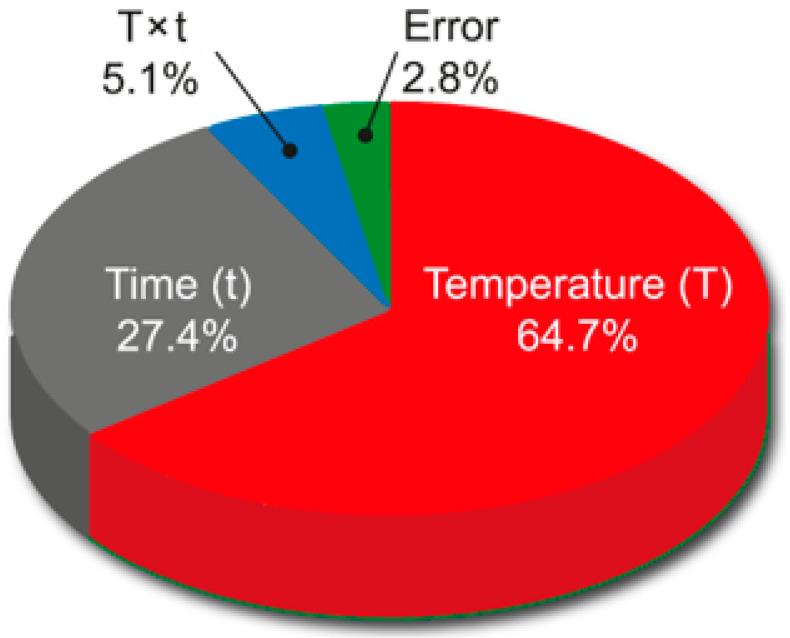
Pie chart illustrating the contribution of the different factors in the analysis of variance (ANOVA) for the regression model.

The results of the ANOVA analysis indicate that the high F-values strongly suggest that the observed differences between the groups are likely to be significant rather than the result of chance. A predefined threshold of statistical significance α = 0.05 was compared with the *p*-value results, revealing that all values fall below the threshold. Consequently, it is concluded that the likelihood of the differences being due to chance is minimal. The results indicate that the null hypothesis H_0_ must be rejected and, in this context, the alternative hypothesis H_1_ must be accepted. The factors were found to be statistically significant in the response, allowing for the determination of the contribution of each factor. Specifically, temperature contributes 64.68%, treatment time 27.37%, and the *t* × *T* combination 5.13%, with an error of 2.82% in the Fe_2_B coating thickness (refer to Figure 23).

Based on the results of the ANOVA analysis of the 4 × 4 factorial design, the software was able to determine a multiple regression model to estimate the Fe_2_B coating thickness with the interactions of temperature (*T*), time (*t*), and combination between both *t* × *T*; the model is expressed as follows:

(38) u(t,T)=−298+0.2798 T−0.02768 t+0.000026 (t×T).

### 4.10. Morphological Analysis of Micrographs

**Figure 24:**
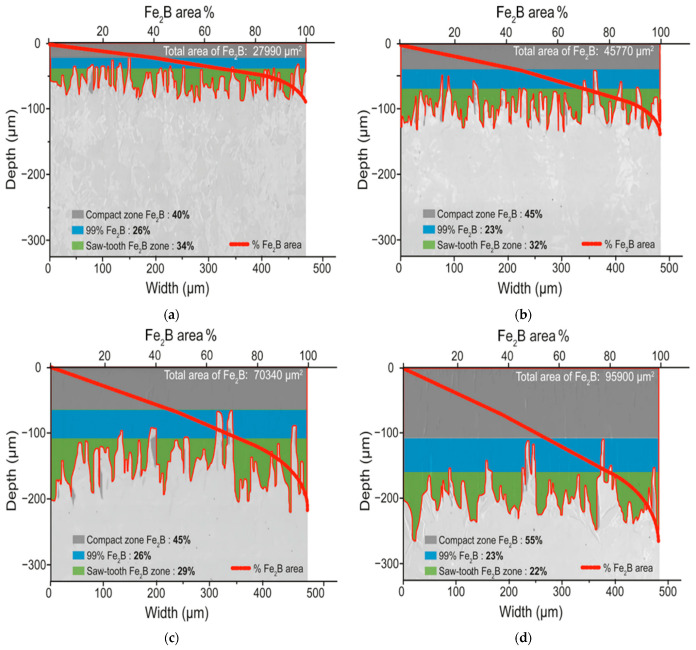
Digital analysis of photomicrographs obtained through Scanning Electron Microscopy (SEM) illustrate the cross-sectional areas of diiron boronized coatings formed on the surface of 35NiCrMo4 steel. The steel was treated at an elevated temperature of 1223 K for varying exposure durations: (**a**) 2 h, (**b**) 4 h, (**c**) 6 h, and (**d**) 8 h.

A detailed digital analysis was conducted using Adobe Photoshop version 24.7 software (San Jose, CA, USA) to analyze the SEM micrographs (refer to Figure 9) depicting the growth fronts of the diiron boronized coating formed on the surface of 35NiCrMo4 steel. The primary objectives were to determine the total area of the Fe_2_B phase and its accumulated area about penetration depth (µm). The analysis revealed a serrated morphology of the Fe_2_B coatings, indicating increased thickness over time and temperature. Subsequently, MATLAB software version R2024a (Natick, MA, USA) was utilized to plot the percentage area of the Fe_2_B phase, with its representation as a red line and corresponding values displayed on the upper axis (refer to Figure 24). 

Moreover, the variation in area distribution with coating depth was observed on the left axis. In contrast, the lower axis provided the width of the analyzed section in micrometers, facilitating a comprehensive understanding of the calculated area. In Figure 24, three characteristic regions of the Fe_2_B coating can be recognized; the first region is colored gray, which is called the compact zone of the Fe_2_B phase and occupies the entire surface; in this region, the Fe_2_B area % curve grows proportionally to the depth. The second detected zone is illuminated in blue, and in this one, we can observe the lowest points or valleys of the typical sawtooth of the Fe_2_B phase; however, the Fe_2_B area % curve remains proportional to the depth due to the high concentration of the Fe_2_B phase. The third region identified in green corresponds to the sawtooth morphology. A completely different area distribution is observed in this region since the Fe_2_B area % curve increases its value rapidly as a function of depth.

## 5. Discussions

### 5.1. Evolution of Diiron Boronized Coating

The proposed mass transfer model is a new alternative to study the evolution of the diiron boronized coating by not considering the solution of the second Fick’s law ($\partial C_{Fe_{2}B}(x,t_{u})/\partial t_{u}=D_{Fe_{2}B}\partial^{2}C_{Fe_{2}B}(x,t_{u})/\partial x^{2}$). The boron concentration profile is expressed in a Taylor series, which is an approximation of functions by a sum of integer powers of polynomials, unlike the existing mass transfer models, where they consider the solution of the second Fick’s law without the influence ($C_{Fe_{2}B}(x)=C_{1}x+C_{2}$) [30] and with the influence of time ($C_{Fe_{2}B}(x,t_{u})=A+Berf(x/\sqrt{4D_{Fe2B}t_{u}})$) [31]. Our findings indicate that the estimated value for the dimensionless interfacial constant $\varepsilon_{Fe_{2}B}^{2}=1-\sqrt{1-4\left(C_{up}^{Fe_{2}B}-C_{low}^{Fe_{2}B}/C_{up}^{Fe_{2}B}+C_{low}^{Fe_{2}B}-2C_{0}\right)}/2=9.6\times 10^{-3}$ aligns with other mass transfer models proposed to study the evolution of the diiron boronized coating [11,32]. To elucidate this alignment, we expand the square root term $\sqrt{1-4\left(C_{up}^{Fe_{2}B}-C_{low}^{Fe_{2}B}/C_{up}^{Fe_{2}B}+C_{low}^{Fe_{2}B}-2C_{0}\right)}$ in a Taylor series, considering only up to the first order, to derive $\sqrt{1-4\left(C_{up}^{Fe_{2}B}-C_{low}^{Fe_{2}B}/C_{up}^{Fe_{2}B}+C_{low}^{Fe_{2}B}-2C_{0}\right)}\approx 1-2(C_{up}^{Fe_{2}B}-C_{low}^{Fe_{2}B}/C_{up}^{Fe_{2}B}+C_{low}^{Fe_{2}B}-2C_{0})$. Consequently, Equation (26) can be expressed as $\varepsilon_{Fe_{2}B}^{2}=C_{up}^{Fe_{2}B}-C_{low}^{Fe_{2}B}/C_{up}^{Fe_{2}B}+C_{low}^{Fe_{2}B}-2C_{0}$, aligning with the findings of previous works [11,32]. The disparity in approach and resolution between the diffusion model and other reported models [11,32] notwithstanding, some correspondence exists in the diffusion models for the evolution of the diiron boronized coating. The examination of the evolution of the diiron boronized coatings holds excellent significance in many different metallic alloys, particularly in applications with a drive to enhance the mechanical and tribological properties of metallic alloys. A comprehensive understanding of the evolution of the diiron boronized coatings is imperative for optimizing and automating thermochemical surface hardening processes and monitoring the quality of the resultant coatings and materials possessing specified properties. This understanding is crucial for various applications required in the metalworking industry, e.g., surfaces with increased wear and corrosion resistance. In the last thirty years, the boronizing process has evolved, with a large universe of data on the formation of the diiron boronized coating on the surface of various metal alloys, highlighting artificial neural networks as one of the exponents of artificial intelligence (AI), under the umbrella of machine learning, that can make possible the estimation of the thicknesses of the boronized coatings, which will replace the conventional mass transfer models.

### 5.2. Analysis of the Microstructural Characterization of Boronized Coatings (SEM)

The microphotographs presented in Figure 11 illustrate the cross-sectional areas of the diiron boronized coatings obtained through Scanning Electron Microscopy. By visual inspection, two well-defined regions can be seen: a diiron boronized coating and the substrate at the underlying part. A close inspection of the diiron boronize coatings formed in all the hardened specimens shows that the coatings are homogeneous with a solid and uniform thickness. A sawing morphology is seen at the growth front in the microphotographs shown in Figure 11. The diiron boronize coatings present a tetragonal crystalline structure centered on the body (see Figure 2). From previous studies, it has been found that the iron boronize crystals grow randomly on the surface of the specimen; subsequently, inside the specimen, the crystals grow with a particular inclination but are oriented along the crystallographic plane (002); finally, at the growth front, the density ($\rho_{Fe_{2}B}=$ 77,430 kg∙m^−3^) increases in the crystallographic direction perpendicular to the surface of the specimen. The sawtooth morphology of the growth front of the diiron boronize coatings depends on various factors, including the chemical composition of the metallic alloy, the temperature of the thermochemical surface hardening process, and the exposure time of the process. According to the literature, the growth fronts of the diiron boronize coatings formed in metallic alloys without and of low chemical composition generate a typically sawtooth growth front, which helps with the grip and adhesion of the diiron boronize coatings and the base material. According to the results obtained in Section 4.1, corresponding to the X-ray analysis, only the diiron boronize phase is present. Also, with the help of Image-Pro Plus v.10.0 software, the average thickness of the diiron boronize coating was determined. To ensure that reliable data are available for measuring the thickness of the diiron boronize coating, it is essential to consider several coating segments for a large number of measurements.

### 5.3. Analysis of Non-Destructive Chemical Techniques

After hardening the borated samples through thermochemical treatment, X-ray diffraction analysis (XRD) was performed. This versatile and non-destructive analytical technique was used to identify the phases present and the composition, crystalline structure, and orientation of the specimens hardened through a thermochemical surface hardening process. A single-phase or multiphase structure in the diiron boronize coating can significantly affect its tribological, mechanical, and adhesion properties. According to the diffraction patterns obtained from the three specimens hardened with the highest temperature (1273 K) at three different times (2 h, 6 h, and 8 h), in the results, it was identified that only a single phase was formed after the thermochemical surface hardening process, known as Fe_2_B in a range of diffraction angles from 0 degrees to 120 degrees. Phase identification can be performed by comparing X-ray diffraction patterns obtained from unknown specimens with patterns from reference databases. The International Center for Diffraction Data (ICDD) maintains the most comprehensive composite database. According to ICDD reference number 00-036-1332, the prominent peaks observed at a diffraction angle of 52 degrees correspond to the three highest intensity values for the Fe_2_B phase. The formation of a single-phase phase according to the results observed with the X-ray diffraction analysis is congruent with the results presented in Section 4.3, wherein Figure 11, the cross sections of the SEM microphotographs that integrate it, shows a single-phase microstructure. 

Energy dispersive X-ray spectroscopy (EDS) is an analytical technique that allows for the chemical characterization and elemental analysis of metallic alloys. In Figure 8, two EDS chemical analyses are presented on the cross-sectional area of steel boronized through the thermochemical surface hardening process at the maximum temperature of 1273 K during an exposure time of 8 h. According to the results obtained in Figure 8a,b, which correspond to the analysis in the surface region and near the growth interface, respectively, the position of the peaks in the spectrum identifies the chemical elements, while the intensity of the signal corresponds to the concentration of the elements. It was determined that the boron content in both regions corresponded to a weight percentage of 8.25 and 7.03 weight per cent of boron. Considering the phase diagram in Figure 2 and various studies, the Fe_2_B phase contains 9.0 weight per cent of boron. In comparison, the FeB phase contains 16.4 weight per cent of boron, which allows us to affirm that the results estimated with the EDS technique in the boron composition are close to the theoretical value of 9.0 weight per cent of boron that identifies the Fe_2_B phase occurring on the surface of 35NiCrMo4 steel. The slight difference between the values obtained experimentally and the theoretical value is likely because the chemical element boron is very difficult to detect, particularly the chemical elements with atomic numbers lower than eight. 

### 5.4. Coefficient of Friction Measurement (COF)

A better understanding of material wear processes ensures that mechanical components are designed with an optimum coefficient of friction. In Figure 17, three friction coefficient profiles are presented for three specimens hardened by the thermochemical surface hardening process at the highest temperature of 1273 K for different exposure times ranging from 2 to 8 h and compared with the friction coefficient profile of the untreated specimen. The tests were conducted under dry conditions, i.e., without lubrication and at room temperature. As shown in Figure 17, at the beginning of the tests, the coefficients of friction (COF) increase slightly and then decrease with increasing sliding distance. These performance patterns are typical of COF measurements and can be related to a phenomenon known as running-in. The surface topology tends to change through the run-in, and chemical reactions occur until the system reaches equilibrium or a steady state, which, for our study, is reached in the 200 to 800 m sliding distance range. Likewise, as can be observed in Figure 17, the COF of the untreated specimen is, on average, about 2.2 in magnitude higher than that of the three hardened specimens. On the other hand, the COF presented by the hardened specimens is reduced; this can be related, according to the literature, to the atmospheric conditions [33], i.e., the presence of oxygen, which drives the generation of an oxide film and the combination between iron and boron atoms inside the diiron boronize coating.

### 5.5. Practical Implications of the ANOVA Results

The statistical analysis carried out allowed us to evaluate the statistical significance of the factors of exposure time and temperature on the thickness of the boronized coating formed on the surface of the 35NiCrMo4 steel during the boronizing process. For this purpose, a factorial experiment design and an ANOVA analysis of variance were used to evaluate the primary factors and their interaction. The results showed the percentage contribution of each factor in the observed variability, showing that temperature contributed 64.68%, time 27.37%, and the interaction between time and temperature 5.13%, and the measurement error was 2.82%. Likewise, the F-value and *p*-value were identified, showing that all factors have a statistically relevant impact on the thickness of the boronized coating, with a significance level of α = 0.05. The results of the ANOVA analysis provide us with a simple understanding of how temperature and treatment time affect the boronizing process, which, in turn, has a direct implication in industrial settings since prioritizing treatment temperature and using time as a secondary adjustment can allow the industrial sector of thermochemical treatments to optimize the boronizing process to increase energy efficiency, profitability, and quality control by proposing a regression model (see Equation (38)) that estimates the magnitude of the thickness of the diiron boronize coating as a function of time, boronizing temperature, and their interaction. The recommended coating thicknesses vary based on specific applications [1]. Thin coatings, ranging from 15 to 20 μm, are utilized to safeguard the substrate versus adhesive wear, including phenomena such as spalling and chip formation, particularly in metal stamping dies and tools. Conversely, approximately 90 to 200 μm thicker coatings are recommended to mitigate abrasive wear, especially in extrusion tooling for plastics subjected to abrasive loads and press tooling within the ceramic industry. For metallic and low-alloying-element alloys, the boronized coating’s optimal thickness range is 50 to 250 μm. In contrast, high-carbon and high-alloy steels exhibit an optimal range of 25 to 76 μm for the same coating.

## 6. Conclusions

The main objective of this work was to study the evolution of the diiron boronize coatings and increase the surface properties of 35NiCrMo4 steel, commonly used in the manufacture of mechanical parts of machinery in general, such as fasteners and motion transmission elements (shafts, shafts, low-speed gears, worm gears, worms, catharines, bolts, high-strength bolts, machine bodies, torsion bars, etc.), using boronizing treatment. The diiron boronize coatings created on the surface of 35NiCrMo4 steel boronized with a commercial boron-rich mixture were examined in detail regarding microstructural, chemical, mechanical, and tribological properties. The thermochemical surface hardening processes were carried out at boronizing temperatures between 1123 and 1273 K, with 2, 4, 6, and 8 h of thermochemical surface hardening processes. The most important results are listed below:

- The boronizing process formed a diiron boronize phase with a particular saw-tooth morphology uniformly distributed on the substrate material’s surface. The specimen boronized at 1273 K for 8 h exhibited the most significant boronize coating thickness. It was shown that the thickness of the diiron boronize coating gradually increased with higher temperatures and longer processing times. Analysis using XRD and EDS confirmed the diiron boronize coating as the only coating present.
- It was shown that the growth kinetics of the diiron boronized coating obey the classical parabolic growth law $u^{2}=4\varepsilon_{Fe_{2}B}^{2}D_{Fe_{2}B}(t_{u}-t_{0}^{Fe_{2}B})$.
- The potential energy barrier for the evolution of the diiron boronized coating was estimated to be about 193 kJ·mol^−1^ for the new diffusion mathematical model.
- Based on the findings illustrated in Figure 17, the wear test results indicate that the COF for the untreated specimen was, on average, around 2.2 times more than the boronized specimen with the thermochemical surface hardening process.
- A novel mathematical model for mass transfer is proposed to study the evolution of the diiron boronize coating. The findings indicate the existence of a dimensionless constant ($\varepsilon_{Fe_{2}B}^{2}=9.6\times 10^{-3}$) consistent with similar constants in diffusion models documented in the existing literature, suggesting the equivalence of these models.
- The average indentation hardness (H_IT_ = 21.59 GPa) and average indentation Young’s modulus (E_IT_ = 349 GPa) were measured on the surface of the diiron boronize coating.
- Two additional parameters (1323 K for 1.5 h and 2.5 h) were used to validate the mathematical model of diffusion. In accordance with Table 3, the estimated coating thicknesses agreed with the experimental data.
- As the ANOVA analysis results showed, each factor contributed to the boronizing treatment: temperature contributed 64.68%, treatment time 27.37%, and the *t* × *T* combination 5.13%, and there was an error of 2.82% in the Fe_2_B coating thickness (see Figure 23).

The boronizing process has led to notable enhancements in the mechanical and tribological properties of the 35NiCrMo4 material, which finds application in the fabrication of shafts, speed gears, worm gears, catarins, bolts, high-strength fasteners, machine bodies, and torsion bars. This improvement holds the potential to maximize the durability of mechanical components made of a metal alloy. Over the past few decades, the boronizing process has witnessed significant progress and is expected to evolve in various aspects. One such direction involves comprehending the growth kinetics through alternative mathematical models, such as artificial intelligence. This promises to stretch the limits to maximize the benefits of the proposed mathematical mass transfer model. Optimizing and automating the thermochemical surface hardening process is essential to lowering production costs and ensuring constant production.

## Figures and Tables

**Table 1:** The experimental data obtained from the plot of squared thickness versus formation time of the diiron boronized phase.

Processing Temperatures	Constant	Kinetic Constant of Growth	Diffusion Coefficient	Typical Incubation Time
T	$\varepsilon_{Fe_{2}B}^{2}$	$4\varepsilon_{Fe_{2}B}^{2}D_{Fe_{2}B}$	$D_{Fe_{2}B}$	$t_{0}^{Fe_{2}B}$
(K)	(Without Units)	(m^2^⸱s^−1^)	(m^2^⸱s^−1^)	(s)
1123	9.6309931 × 10^−3^	1.5019 × 10^−13^	3.8988 × 10^−12^	2052.9652
1173		3.6266 × 10^−13^	9.4140 × 10^−12^	2052.9889
1223		8.1478 × 10^−13^	2.1150 × 10^−11^	2052.9943
1273		1.7177 × 10^−12^	4.4590 × 10^−11^	2052.9487

**Table 2:** The potential energy barrier of boron in diiron boronized coating values is based on this work with data from the literature.

Substrate	Potential Energy Barrier *Q* (kJ·mol^−1^)	Approach	Study
Hardox-450 steel	157.9	Experimental law of increasing thickness	[20]
AISI 9840 steel	193.08	Mass transfer model	[28]
AISI 1018 steel	91.20–155.2	Experimental law of increasing thickness	[25]
Aleación Nimonic 80A steel	190.93	Mass transfer model	[26]
X65Cr14 martensitic steel	206.53	Experimental law of increasing thickness	[23]
AISI P20 steel	200.0	Experimental law of increasing thickness	[27]
SAE 1020 steel	183.15	Experimental law of increasing thickness	[24]
35NiCrMo4 steel	193.0	Mass transfer model	This study

**Table 3:** Data collected for induced coating thicknesses at 1323 K for 1.5 h and 2.5 h compared with the values calculated with Equation (25) for $C_{up}^{Fe_{2}B}$ = 9 wt.% B.

Processing Temperature	Treatment Time	Experimental Coating Thicknesses	Coating Thicknesses Calculated with Equation (25)
T (K)	(s)	(μm)	(μm)
1323	5400	102.3 ± 12.9	107.28
	9000	151.7 ± 19.2	154.55

**Table 4:** Analysis of variance for the Fe_2_B coating thicknesses of boronized 35NiCrMo4 steel.

Boronized Steel	Factor	Degrees of Freedom	Sum of Squares	Principal Squares	F-Value	Contribution	*p*-Value
35NiCrMo4	Temperature (*T*)	3	56,802	18934	122.36	64.68%	0
	Time (*t*)	3	24,040	8013.3	51.79	27.37%	0
	*t* × *T*	9	4504	500.4	3.23	5.13%	0.02
	Error Total	16	2476	154.7	-	2.82%	-
		31	87,821	-	-	100%	-

## Data Availability

The original contributions presented in the study are included in the article, further inquiries can be directed to the corresponding author.
